# Extreme Temperature Events in Kazakhstan and Their Impacts on Public Health and Energy Demand

**DOI:** 10.1002/gch2.202400207

**Published:** 2024-12-19

**Authors:** Parya Broomandi, Alfrendo Satyanaga, Mehdi Bagheri, Mostafa Hadei, David Galán‐Madruga, Ali Mozhdehi Fard, Adib Roshani, Aram Fathian, Amir Nourian, Michael Leuchner, Klaus Reicherter, Mehdi Hamidi, Prashant Kumar, Jong Ryeol Kim

**Affiliations:** ^1^ Department of Civil and Environmental Engineering School of Engineering and Digital Sciences Nazarbayev University Kabanbay Batyr Ave. 53 Astana 010000 Kazakhstan; ^2^ Department of Electrical and Computer Engineering School of Engineering and Digital Sciences Nazarbayev University Kabanbay Batyr Ave. 53 Astana 010000 Kazakhstan; ^3^ Neotectonics and Natural Hazards Institute RWTH Aachen University 52056 Aachen Germany; ^4^ Department of Health in Emergencies and Disasters Tehran University of Medical Sciences Tehran 1417613151 Iran; ^5^ Climate Change and Health Research Center (CCHRC) Institute for Environmental Research (IER) Tehran University of Medical Sciences Tehran 1439813118 Iran; ^6^ Department of Atmospheric Pollution National Centre for Environment Health Health Institute Carlos III. Ctra. Majadahonda a Pozuelo km 2.2 Madrid 28220 Spain; ^7^ Faculty of Civil Engineering Babol Noshirvani University of Technology Babol 484 Iran; ^8^ UNESCO Chair on Coastal Geo‐Hazard Analysis Research Institute for Earth Sciences Tehran 13185‐1494 Iran; ^9^ Water Sediment Hazards and Earth‐surface Dynamics (waterSHED) Lab Department of Geoscience University of Calgary Calgary Alberta T2N 1N4 Canada; ^10^ School of Science Engineering and Environment (SEE) University of Salford, Salford Manchester M5 4WT UK; ^11^ Physical Geography and Climatology Department of Geography RWTH Aachen University Wüllnerstr. 5b 52062 Aachen Germany; ^12^ Institute for Geophysics and Meteorology University of Cologne Pohligstr.3 50969 Cologne Germany; ^13^ Global Centre for Clean Air Research (GCARE) School of Sustainability Civil and Environmental Engineering Faculty of Engineering and Physical Sciences University of Surrey Guildford Surrey GU2 7XH UK; ^14^ Institute for Sustainability University of Surrey Guildford Surrey GU2 7XH UK

**Keywords:** carbon footprint, mann‐kendall trend analysis, renewable energy, sustainable urban development goals

## Abstract

Extreme temperature events such as heatwaves are becoming increasingly severe and frequent because of climate change, posing significant challenges to public health and energy infrastructure. This study explores the impacts of extreme temperature events leading to heat‐/cold waves on public health and energy consumption in Kazakhstan from 1959 to 2021. The most striking trends in heatwave‐related indices emerge in the western and southwestern regions. Conversely, despite heightened coldwave intensity, a decline is noted in their frequency and number. The impact of heatwaves on various health conditions, notably consistent and statistically significant rises in all‐cause and cardiovascular mortalities, is observed. Shifts in energy demand are also unveiled with a noticeable spike in cooling‐degree days and a reduction in heating‐degree days. The mean total energy consumption stood at 552 kWh across the country with an average annual energy generation of ≈8.76 kWh. To gauge the environmental implications, the mean CO_2_ emissions are estimated at 464 kg per kWh for both heating and cooling purposes. With climate change set to escalate heatwaves, the need for comprehensive health planning is underscored to mitigate their adverse health impacts. Furthermore, transitioning from fossil fuels to green energy sources is crucial to reduce the environmental footprint.

## Graphical abstract

This study examines the effects of extreme temperature events, including heatwaves and cold waves, on public health and energy consumption in Kazakhstan from 1959 to 2021. Findings highlight increased heatwave‐related health risks, particularly in western regions, along with shifts in energy demand. The study underscores the need for health planning and a transition to green energy to address these climate‐related challenges.

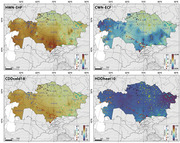

## 1 Introduction

Human activities have significantly affected the atmosphere, resulting in extreme weather events and climate changes. According to the Intergovernmental Panel on Climate Change report, the average global surface temperature was found to be 1.09 °C higher during the decade of 2011–2020 compared to the period from 1850 to 1900. This increase was more significant over land (1.59 °C) compared to the ocean (0.88 °C).^[^ ^1^ ^]^ This increase is largely attributed to human influence and amounts to ≈1.07 °C from 1850 to 2019.^[^ ^1^, ^2^ ^]^ The past three decades are likely the warmest in the Northern Hemisphere over the past 1400 years, and projections indicate further temperature increases throughout the 21^st^ century.^[^ ^3^ ^]^

There is a lack of alignment and universal agreement over the precise definition of heat‐/cold waves (hereafter referred to as heat‐/cold waves). Predetermined absolute values and variances from typical circumstances are frequently employed in different studies. The definition of a heatwave, as provided by the World Meteorological Organization (WMO), is an atypical period of exceptionally high/low temperatures that persists for a minimum duration of three consecutive days, considering into account the prevailing climatic conditions in the local area and beyond predetermined thermal thresholds.^[^ ^1^, ^2^ ^]^

Heat‐/cold waves have been found to have negative impacts on various aspects including human well‐being, air quality, energy supply, and infrastructure.^[^ ^1^, ^4^, ^5^, ^6^ ^]^ Empirical evidence indicates a significant rise in fatalities caused by heat‐related incidents, hence establishing heatwaves as a rapidly expanding category within the spectrum of climate‐related disasters.^[^ ^7^ ^]^ Conversely, cold temperatures have various health consequences, encompassing immediate risks like frostbite and hypothermia. Additionally, cold temperature elevates the likelihood of mortality and hospitalization caused by respiratory and cardiovascular health problems.^[^ ^8^, ^9^, ^10^, ^11^ ^]^ A conducted study in the USA showed that colder weather substantially heightens health risks in Texas, with mortality increasing by 0.1–5.0% and emergency hospital admissions rising by 0.1–3.8% for each 10 °C decrease below cold thresholds.^[^ ^8^ ^]^

Extreme weather events, particularly heat‐/coldwaves, pose significant risks to energy production and consumption, stressing electricity generation and power grid systems and often leading to blackouts. The energy sector faces a dual challenge: it is both vulnerable to extreme weather and a major contributor to it, primarily through fossil fuel‐based power generation, which has been a significant source of greenhouse gas emissions since the early 20^th^ century. This vulnerability to extreme weather introduces unpredictability in power generation and consumption, price volatility, and risks to energy security. For instance, a heatwave can hinder electricity generation in a nuclear plant while simultaneously increasing energy demand due to air conditioning.^[^ ^12^, ^13^, ^14^ ^]^

Furthermore, elevated energy consumption during periods of extreme heat and cold has a direct impact on the elevation of tropospheric ozone inside metropolitan regions. This phenomenon not only raises health‐related issues but also serves to reinforce the urban heat island effect; hence exacerbating the overall energy consumption levels experienced.^[^ ^13^, ^15^ ^]^

Central Asia, located in the hinterland of the Eurasian continent, is characterized as a semi‐arid and arid region facing numerous environmental challenges, including land degradation, water scarcity, and limited emergency management capabilities. These challenges render the region particularly vulnerable to natural hazards such as heatwaves.^[^ ^16^, ^17^, ^18^, ^19^ ^]^ During the period from 1990 to 2010, extreme temperature events in Central Asia resulted in substantial economic losses of approximately US$1 billion, ranking them among the most significant meteorological disasters in terms of economic impact.^[^ ^20^ ^]^

Kazakhstan, located in the northern part of Central Asia, has experienced more rapid climate changes compared to other regions within the same latitudinal zone. From 1950 to 2020, average air temperature in Kazakhstan increased at a rate of 0.31 °C per decade, based on measurements from 110 weather stations.^[^ ^19^ ^]^ The most pronounced temperature rise occurred during the last two decades, while no significant trends in precipitation were observed.^[^ ^18^, ^19^ ^]^

Despite these documented climatic changes, there remains a significant lack of comprehensive studies addressing the impacts of temperature extremes in Central Asia. Recent reviews of related research highlighted several critical gaps, including an overemphasis on the causes, effects, and mitigation of urban heat, while largely neglecting adaptation and preparedness measures.^[^ ^21^ ^]^ Additionally, there is an incomplete understanding of heat‐related impacts, a focus on microclimate and heat islands rather than extreme heat, and insufficient integration of social, economic, and policy support. Moreover, the challenges in addressing these gaps include limited resources for research, insufficient collaboration among different disciplines, and inadequate policy frameworks to support heat adaptation efforts.^[^ ^21^ ^]^ These gaps are particularly concerning, as urban areas in Kazakhstan are increasingly experiencing severe temperature extremes, exacerbated by urban heat effects.

Given the potential impacts of temperature extremes on human health and energy demand, and the projected increase in frequency, duration, and intensity of these extreme events,^[^ ^21^ ^]^ it is imperative to thoroughly investigate heat‐/coldwaves in Kazakhstan. The urban heat island effect may also play a role, as certain areas in Kazakhstan may be undergoing increasing urbanization, potentially resulting in cities that can be several degrees Celsius warmer than surrounding rural areas. This differentiation between urban and rural temperatures is critical for accurately identifying heat and cold prone zones and for developing appropriate adaptation measures.^[^ ^21^ ^]^ This study aims to contribute to the understanding of the considerable spatial and temporal variability of heat‐/coldwaves, their socioeconomic impacts, and the limited research on these phenomena in both urban and rural contexts.

The present study aims to achieve four main objectives: (1) evaluate the spatiotemporal distribution of heat‐/coldwaves to identify areas highly vulnerable to these extremes; (2) analyze the spatiotemporal distribution of both hot and cold days and nights; (3) assess the health impacts associated with heat‐/coldwaves, including their temporal distribution; and (4) assess the energy demand associated with heat‐/coldwaves, considering their spatial and temporal distribution across Kazakhstan from 1959 to 2021. This study ultimately aims to provide valuable insights into the dynamics of extreme temperatures in Kazakhstan, thereby supporting informed decision‐making and the development of effective mitigation strategies to address the challenges posed by heat‐/cold extremes, with an emphasis on the urgent need for adaptation measures.

## 2 Methodology

### 2.1 Study Domain

**Figure 1:**
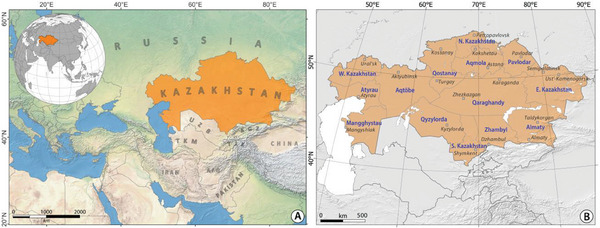
A physiographic map of Kazakhstan, Central Asia (study area).

Kazakhstan in the Central Asia (**Figure** **1**), ranks as the ninth‐largest country globally, covering ≈2.7 million square kilometers.^[^ ^19^ ^]^ The majority of its territory consists of plains and lowlands, with mountainous regions mainly found in the eastern and southeastern areas.^[^ ^22^ ^]^ As part of the Asian Arid Zone, Kazakhstan experiences high risk of droughts and limited precipitation. Over the past century, the Asian Arid Zone has demonstrated high sensitivity to climate change and is anticipated to face significant impacts from projected future warming.^[^ ^23^, ^24^ ^]^ Kazakhstan's geographical location at the heart of Asia results in minimal influence from oceans on its climate. Air masses from the Pacific and Indian oceans have little penetration into the country, while the Atlantic Ocean significantly influences the regional climate through the transportation of moist air masses.^[^ ^19^, ^25^ ^]^

The climate in Kazakhstan is predominantly continental in nature. Annual precipitation varies between 250 and 350 mm in the northern regions to merely 100 to 120 mm in the southern areas. January temperatures average ≈−15 °C, with minimum values plummeting to −40 °C.^[^ ^19^, ^25^ ^]^ The summers are characterized by relatively hot conditions, with mean July temperatures reaching up to 40 °C in desert steppes and low‐lying steppes.^[^ ^19^, ^25^ ^]^

Based on the Köppen‐Geiger climate classification, the eastern and northern parts of Kazakhstan fall under cold climates with hot summers.^[^ ^26^ ^]^ On the other hand, the southern and western regions experience dry steppe and desert climates.^[^ ^27^ ^]^

Moreover, Kazakhstan has also experienced notable urbanization trends over the past decade. Between 2009 and 2019, the urban population grew from 8.5 million to 10.5 million, representing a growth of 23%.^[^ ^28^, ^29^ ^]^ The share of the population living in urban areas increased from 53.2% in 2009 to 57.6% in 2019. Major cities such as Almaty, Astana, and Shymkent have seen substantial growth, with Almaty's population rising from 1.36 million to 1.85 million and Astana's population growing from 605 000 to over 1 million during this period. Shymkent also experienced significant urban growth, with its population surpassing 1 million by 2019. This urban expansion is primarily driven by socio‐economic opportunities in urban areas, including better access to jobs, education, and healthcare, attracting people from rural regions. The urbanization process in Kazakhstan highlights a shifting demographic pattern, which has implications for infrastructure, resource allocation, and climate adaptation strategies.^[^ ^28^, ^29^ ^]^

### 2.2 Data

In the current research, the initial meteorological data were derived from daily‐based ERA5 (ECMWF Reanalysis v5) reanalysis data, which include the average daily air temperature, their extreme values (minimum and maximum), and daily atmospheric precipitation sums from a total of 320 locations across Kazakhstan.^[^ ^30^ ^]^ These locations comprise cities, towns, and villages. The data covers the time period from 1959 to 2021 and serves as a comprehensive source for our analysis.

Moreover, the spatial representation and interpolation methods were performed employing the QGIS software, Version 3.22.3.^[^ ^31^ ^]^ We utilized the interpolation technique of the Inverse Distance Weighting (IDW) algorithm to generate a raster output with a spatial resolution of 0.1° by 0.1°.

### 2.3 Clustering Using K‐Means Method

To investigate the possible differences in climate indices in various regions within Kazakhstan, we decided to group them into clusters based on their maximum and minimum temperatures, total precipitation, and geographical characteristics. One widely used clustering method is the K‐means algorithm, wherein samples are grouped into groups based on similar characteristics.^[^ ^32^ ^]^ In this approach, the number of clusters is pre‐established, and the validation of the suitable number of clusters was conducted using the Elbow index, which was computed using Equation (1) as proposed by Brusco and Steinley.^[^ ^33^ ^]^

(1) $$WCSS={\sum_{k=1}^{K}\sum_{i\in C_{k}}\sum_{v=1}^{V}\left(x_{iv}-\bar{x_{vk}}\right)}^{2}$$

The sets of data in the Kth cluster are denoted as *C_k_*, and the mean of the variable *v* inside the cluster is represented as $\bar{x_{vk}}$. To ascertain the optimal number of clusters, a graphical representation is constructed wherein the horizontal axis denotes the number of clusters, while the vertical axis is represented by *WCSS*. The computation of the value of *K* is performed for different values of, commencing from 1 and progressively increasing until the point at which the value of *WCSS*stabilizes or reaches a plateau‐like state, typically corresponding to the maximum number of clusters. The point on the plot commonly referred to as the “Elbow” point is typically seen as indicative of the ideal number of clusters for the given data.^[^ ^34^, ^35^ ^]^

### 2.4 Calculation of Climate Indices

To monitor the extreme climatic conditions, the suggested climatic indices by the WMO climatology commission^[^ ^36^ ^]^ were employed. The software package ClimPACT2 (https://climpact‐sci.org) was utilized for the calculation and evaluation of these indices, facilitating the examination of extreme climate events and their temporal variations.

The least squares method was employed to compute linear trends in determining the long‐term trends in precipitation and air temperature patterns. The Mann‐Kendall nonparametric test was used to assess the statistical significance of the observed trends.^[^ ^25^, ^37^, ^38^, ^39^ ^]^

#### 2.4.1 Heat‐/Coldwave Indices

In the current work, the yearly values of the heatwave indices, including heatwave number (HWN), heatwave frequency (HWF), heatwave amplitude (HWA), heatwave duration (HWD), and heatwave magnitude (HWM) were calculated. Three distinct heatwave definitions were employed using EHF (excess heat factor), T_max_ (maximum temperature), and T_min_ (minimum temperature) as explained in Supplementary material. Conversely, cold waves refer to periods of unusually cold temperatures, which are determined in ClimPACT2 using the Excess Cold Factor (ECF),^[^ ^40^ ^]^ as explained in SI.

Moreover, we examined the percentage of cool days (TX10p) and nights (TN10p), warm days (TX90p) and nights (TN90p), consecutive dry days (CDD), cooling‐degree days (CDDcold18), and heating‐degree days (HDDheat10).^[^ ^41^ ^]^ Table S1, Supporting Information illustrates all the climate indices, which were utilized to categorize extremities into four conditional categories:

- Event duration‐based indices.
- Thresholds‐based indices.
- Indices based on percentile.
- Absolute value‐based indices.

### 2.5 Health Impact Assessment

We evaluated the health impacts of heatwaves using the methodology established by Nori‐Sarma et al.^[^ ^42^ ^]^ This process determines the number of heatwave‐related deaths by first identifying the attributable risk percentage, which signifies the percentage of total deaths directly linked to heatwaves. This percentage is found using relative risk estimates. The daily average of expected community deaths and the number of heatwave days are then factored in, and the attributable risk percentage is applied to the resulting figure. This calculation produces the total number of deaths per heatwave definition attributable to the incidence of heatwaves, as outlined in Equation (2):^[^ ^42^ ^]^

(2) $$\begin{array}{c} \mathrm{Attributable}\;\mathrm{mortality}=\mathrm{heatwave}\;\mathrm{days}\times death_{daily}\times \frac{\left(RR-1\right)}{RR} \end{array}$$

Here, *heatwave days* refer to the annual number of days classified as heatwaves, *death_daily_* represents the daily community death count, and *RR* is the relative risk of heatwave effects on mortality, obtained from epidemiological studies.

Data for all‐cause mortality during the period 1959 to 2021 was sourced from the online database https://macrotrends.net. Numbers for cardiovascular (CVD) deaths were obtained from the Global Burden of Disease Collaborative Network (available at: https://vizhub.healthdata.org/gbd‐results), spanning the years 1990 to 2019.

In the absence of daily mortality data in Kazakhstan and Central Asia, daily mortality rates were estimated by dividing the annual mortality totals by the number of days in a year (365 or 366). While this estimation approach is simplified, it is a standardized method commonly employed in regions with limited data availability, providing a consistent basis for assessing daily mortality across the study.^[^ ^43^, ^44^ ^]^

Despite this methodological limitation, the analysis maintains robustness through the application of global meta‐analyses. These meta‐analyses synthesize data from a wide range of studies to produce comprehensive and statistically reliable estimates of relative risk (RR), a crucial metric for evaluating the health impacts of temperature extremes. This approach is widely recognized in regions where local epidemiological studies are unavailable, allowing us to utilize the best available evidence to inform risk estimates.^[^ ^45^, ^46^ ^]^ Relative risks for all‐cause mortality due to heatwaves were derived from global meta‐analyses, yielding values of 1.20 (95% CI: 1.02, 1.41) from Xu et al.^[^ ^46^ ^]^ and 1.04 (95% CI: 1.03, 1.06) for general mortality. Cardiovascular‐specific relative risks were obtained from Cheng et al.,^[^ ^45^ ^]^ reporting a value of 1.15 (95% CI: 1.09, 1.21). However, in the absence of meta‐analyses or localized studies examining the health impacts of coldwaves in Kazakhstan and Central Asia, a comprehensive health risk assessment for coldwaves could not be undertaken.

By employing established, peer‐reviewed data sources and maintaining a consistent methodological framework throughout the analysis, the credibility and reliability of the findings are ensured. The standardized approach, applied both in the estimation of daily mortality and in the calculation of relative risks, enhances the comparability and accuracy of results, making them applicable to regions where local epidemiological data is scarce.

### 2.6 Energy Demand for Cooling and Heating Needs and Associated CO_2_ Emissions

The degree‐day approach was used in assessing energy demands for cooling and heating at both individual building and regional housing stock levels.^[^ ^47^, ^48^ ^]^ This approach allows for a comprehensive estimation of energy requirements, reflecting the varied energy use patterns and thermal properties of different building types as they adapt to climate change.

**Figure 2:**
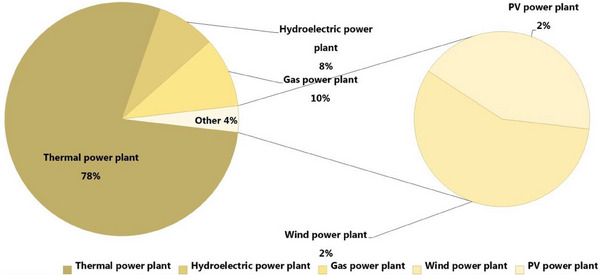
The configuration of energy supply in Kazakhstan (Data:^[^ ^5^ ^]^).

Additionally, taking into account the configuration of the energy generation system in Kazakhstan (https://www.kegoc.kz), and given that the predominant source of energy in Kazakhstan is derived from thermal power plants, an evaluation of the amount of CO_2_ emissions produced per unit of energy consumption was conducted. This assessment utilized the data presented in **Table** **1** and **Figure** **2**.^[^ ^49^ ^]^

**Table 1:** Carbon Footprint of Different Fossil Fuels and Renewable Energy Sources for Producing 1 kWh of Electricity.^[^ ^49^ ^]^

Fuel Type	CO_2_ Footprint (gr)
Coal‐fired plant	960
Gas‐fired plant	869
Oil‐fired plant	596
Combined‐cycle gas	450
Hydroelectric	4
PV	100
Wind	15

Furthermore, given that building heat loss rates can fluctuate based on stricter building efficiency regulations and evolving household expectations for thermal comfort, our approach involves calculating energy demands per unit of heat loss (heating purpose) and thermal transmittance per square meter (cooling purpose) independently of building types and efficiency standards.

#### 2.6.1 Energy Generation for Heating

The required energy for heating purposes in a building is directly linked to the building's heat losses, which can fluctuate based on the changes in the temperature difference between the outdoor and indoor environments (considering factors like air infiltration, natural ventilation, thermal radiation losses, and wall heat conduction).^[^ ^47^ ^]^

As a result, the total energy demand for space heating during the specified time frame, denoted as *Q*, can be calculated using Equation (3):

(3) Q=P×HDD

where *HDD* and *P* are heating‐degree days and heat loss rate (KWh °C^−1^), respectively.

#### 2.6.2 Energy Generation for Cooling

The total cooling load needed is determined using Equation (4):^[^ ^48^ ^]^

(4) Q=CU×CDD

where *CDD*, U‐value, and *C* are cooling‐degree days, thermal transmittance or heat transfer coefficient (KWh m^−2^ °C), and the area of the building (m^2^), respectively. The base temperatures for cooling and heating purposes were set to 18 and 10 °C following ASHRAE standards.^[^ ^50^, ^51^ ^]^

## 3 Results and Discussion

Kazakhstan was divided into seven clusters to analyze the spatial distribution of climatic indices (**Table** **2**). K‐means clustering method was used to cluster considering the precipitation, temperature at 2‐m (minimum and maximum). The ranges of average precipitation (summation between 1959 and 2021), minimum temperature at 2‐m (averaged between 1959 and 2021), and maximum temperature at 2‐m (averaged between 1959 and 2021) for each identified cluster are presented in Table 2.

**Table 2:** Main clusters of Kazakhstan in the current study between 1959 and 2021(It worth mentioning that precipitation is summed over the whole period of study).

Cluster	Region	Cluster No.	Maximum 2‐m temperature [°C]	Minimum 2‐m temperature [°C]	Precipitation [mm]
North Kazakhstan	Qostanay	**1**	3.3–4.8	2.8–4.2	224 358–30 232
Northwest Kazakhstan	Aqtobe	**2**	4.7–9.3	4.1–8.6	10 906–30 683
Northeast Kazakhstan	North Kazakhstan, Pavlodar, Aqmola, Garaghandy	**5**	−0.3–5.8	−1.0–5.1	20 523–83 414
West Kazakhstan	West Kazakhstan, Atyrau, Mangystau	**6**	0.4–13.6	−0.5–13.1	10 064–77 602
East Kazakhstan	East Kazakhstan	**3**	2.7–6.7	2.1–6.0	14 386–31 424
Southeast Kazakhstan	Almaty, Zhambyl	**4**	6.5–14.2	5.9–13.6	10 642–28 143
South Kazakhstan	Gyzylorda, South Kazakhstan	**7**	7.7–15.9	7.0–15.1	6720–60 991

### 3.1 Spatial and Temporal Variations in the Climate Indices in Kazakhstan

#### 3.1.1 Changing Patterns in the Percentage of Cool and Warm Days and Nights in Kazakhstan

**Figure 3:**
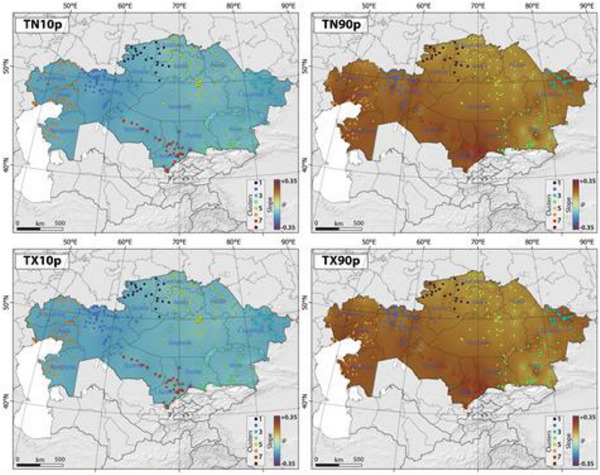
The slope of spatial‐temporal changes in the TN10p, TN90p, TX10p, and TX90p in Kazakhstan, Central Asia, between 1959 and 2021. Please note that colored dots and color scale refer to identified clusters and slope of changes, respectively, in Kazakhstan.

Figure 2 illustrates the spatial and temporal distribution of the TX10p, TX90p, TN10p, and TN90p. Our findings reveal a notable and statistically significant decline in the proportion of cool days (A) and cool nights (C), alongside a statistically significant rise in the proportion of warm days (B) and warm nights (D) (**Figure** **3**).

Analyzing the trends in TN10p and TX90p across the map has enabled the distinction of Kazakhstan's territory into two distinct halves based on the extent of climate change. The southwestern half shows a notable trend toward higher extreme daytime temperatures as well as lower extreme nighttime temperatures. Conversely, the northeastern part displays a comparatively less pronounced trend, both in terms of extremely low nighttime temperatures and high daytime temperatures.

Our findings indicated that the most significant decreases in the percentage of cool nights and days were observed in west Kazakhstan, northwest Kazakhstan, and east Kazakhstan. Similarly, warm nights and days exhibited more pronounced increases over time in West Kazakhstan, northwest Kazakhstan, east Kazakhstan, and south Kazakhstan (Figure 3). In contrast, the least noticeable changes in the percentage of cool nights and days, as well as warm nights and days, were witnessed in north Kazakhstan, northeast Kazakhstan, and southeast Kazakhstan (Figure 3). Table S2, Supporting Information presents the maximum and minimum Sen's slope values, which indicate the most significant and least significant rise or fall in the proportion of cool days and nights, as well as warm days and nights across various clusters in Kazakhstan. Based on our findings, the following cities exhibited the most notable and statistically notable spikes in the percentage of warm days: Komsomolets with a slope of 0.21, Temir with a slope of −0.27, Shar with a slope of 0.23, Saryozek with a slope of 0.34, Dzhambul with a slope of 0.18, Zhanbay with a slope of 0.30, and Taraz with a slope of 0.35 across clusters 1, 2, 3, 4, 5, 6, and 7, respectively. Komsomolets, Temir, Shar, Saryozek, Dzhambul, Zhanbay, and Taraz exhibited a consistent trend of increase in warm nights across clusters 1, 2, 3, 4, 5, 6, and 7, respectively. Conversely, the cities of Aqqaytym (with a slope of −0.16), Taraz (with a slope of −0.16), and Taraz (with a slope of −0.20) exhibited the most significant reductions in the percentage of cool days, cool days, and cool nights, respectively, across different clusters in Kazakhstan.

In a similar study, Salnikov et al.^[^ ^52^ ^]^ indicate the presence of minor and statistically insignificant negative trends in the index of T_X_, representing the highest monthly maximum daily air temperatures in Kazakhstan, mainly in the northern and northeastern regions. Significant positive trends, with values ranging from 0.4 to 0.7 °C per decade, were predominantly recorded in the western regions of Kazakhstan. In contrast, it can be observed that minimum daily air temperatures (T_N_) generally displayed a positive trend. However, it is worth noting that significant trends were primarily evident in southern and central parts of Kazakhstan. Additionally, significant positive trends were also identified in southeastern regions of Kazakhstan and along the coastline of the Caspian Sea.^[^ ^25^, ^52^ ^]^

Moreover, the prevalence of extremely warm nights and hot days did not experience uniform growth across Kazakhstan. The most substantial positive TX90p trends were observed in the central, western, and southern regions (1.4–2.7% per decade), while the northern and eastern areas witnessed values decreasing to 0.4–0.9% per decade. The incidence of warm nights displayed a scattered pattern, with an overall trend ranging between 2.4% and 2.6% per decade. In the remaining areas of Kazakhstan, the trend varied moderately from 0.7–1.5% per decade. Conversely, the percentage of cold days and nights diminished from the northeast to the southwest.^[^ ^52^ ^]^

#### 3.1.2 Changing Patterns in Heatwaves across Kazakhstan

**Figure 4:**
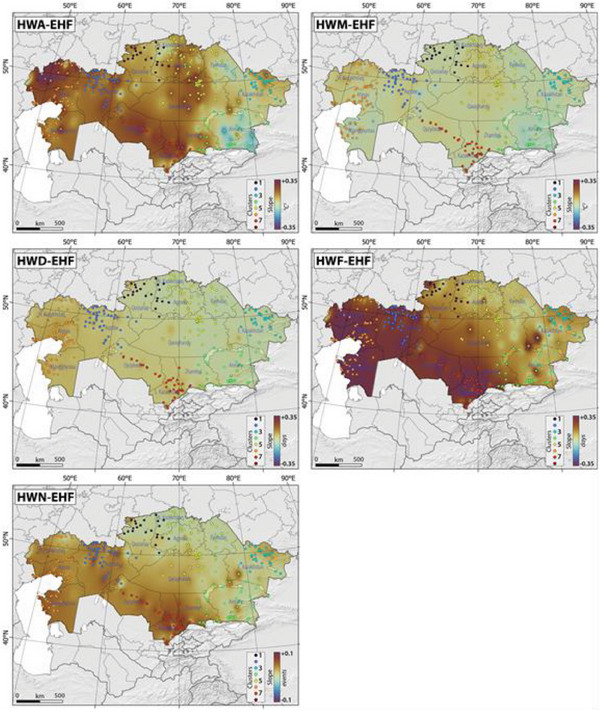
The slope of spatial‐temporal changes in the HWA‐EHF, HWM‐EHF, HWD‐EHF, HWF‐EHF, and HWN‐EHF in Kazakhstan, Central Asia, between 1959 and 2021. Please note that colored dots and color scale refer to identified clusters and slope of changes, respectively, in Kazakhstan.

**Figure** **4** and Figures S1 and S2, Supporting Information illustrate the spatial and temporal distribution of HWA, HWD, HWF, HWM, and HWN as defined by the Excess Heat Factor, the 90^th^ percentile of T_N,_ and the 90^th^ percentile of T_X_.

Our results demonstrate a consistent rise in the indices of HWA, HWD, HWF, HWM, and HWN across Kazakhstan. Notably, statistically significant changes were observed in regions like north Kazakhstan, northwest Kazakhstan, east Kazakhstan, south Kazakhstan, and southeast Kazakhstan (Figure 4 and Figures S1 and S2, Supporting Information). Conversely, east Kazakhstan exhibited relatively lower vulnerability, with a more stable trend in heatwave indices. The amplitude and frequency of heatwaves exhibited a significant rise throughout most of Kazakhstan, with exceptions in certain areas of southeast Kazakhstan (Figure 4 and Figures S1 and S2, Supporting Information), resulting in a higher number of days characterized by the highest mean temperature among all heatwave occurrences. Meanwhile, the duration and number of heatwaves notably increased over the years in regions like northwest Kazakhstan, west Kazakhstan, and south Kazakhstan (Figure 4 and Figures S1 and S2, Supporting Information).

The magnitude of heatwaves experienced a notable surge in northwest Kazakhstan and certain parts of northeast Kazakhstan, while certain sections of southeast and east Kazakhstan displayed a decrease in heatwave magnitude (Figure 4 and Figures S1 and S2, Supporting Information). Table S2, Supporting Information presents the Mann‐Kendall trend analysis results for variables TX10p, TX90p, TN10p, and TN90p across various clusters in Kazakhstan from 1959 to 2021, revealing notable increases in the percentage of warm days and nights despite significant declines in cool days and nights. Additionally, Table S3, Supporting Information details the highest values of Sen's slope, indicating the most significant increases in HWN, HWD, HWM, HWF, and HWA based on the Excess Heat Factor across different clusters in Kazakhstan. Our findings highlight that Cluster 7, Karatau (with a slope of 0.399), Cluster 6, Mikil (with a slope of 0.167), Cluster 7, Taraz (with a slope of 0.577), Cluster 6, Tasqala (with a slope of 0.112), and Cluster 4, Saryozek (with a slope of 0.096) experienced the most significant changes in terms of HWA, HWD, HWF, HWM, and HWN. Our findings also revealed certain parallels between the shifts in heatwave indices and the proportions of cool and warm days and nights. For instance, Taraz in cluster 7, which exhibited the most significant alterations in HWF and HWN, also demonstrated the highest rise in the percentage of warm nights and days, coupled with a decline in cool nights and days (Tables S2 and S3, Supporting Information).

Previous studies have also indicated a significant increase in the frequency and length of heatwaves in Central Asia during the 1990s.^[^ ^18^ ^]^ Salnikov et al.^[^ ^52^ ^]^ showed a distinct and statistically significant trend was revealed, indicating a rise in heatwave duration moving from the eastern to the western regions of Kazakhstan.^[^ ^52^ ^]^

These heatwave trends in Central Asia exhibited a strong correlation with a zonal wave circulation pattern at 500 hPa. This circulation pattern was characterized by the presence of positive geopotential height anomalies centered over the central area of Asia, as documented by Yu et al.^[^ ^18^ ^]^ The intensification of this anomalous circulation pattern during the 1990s strongly indicates that larger‐scale atmospheric circulation patterns have played a role in influencing the frequency of heatwaves in the Central Asian region.^[^ ^18^ ^]^

Moreover, in Central Asia the spatial distribution of the trends observed in heatwave indices closely aligned with the patterns of climatic warming trends (T_max_) across the region. This alignment strongly implies a direct influence of large‐scale climate warming on the escalation of heatwave occurrences in the area.^[^ ^5^, ^52^ ^]^

Recently Hantemirov et al.,^[^ ^53^ ^]^ assessed recent temperature changes in Siberia by analyzing tree‐ring data from the Yamal Peninsula in northwestern Siberia. They showed that both the mean June‐July (JJ) air temperatures (12.83 °C) and the rate of warming (0.0173 °C per year) between 1850 and 2019 exceeded the range of natural climate variability in the broader study area.

The above findings allow us to conclude that the rapid observed warming could lead to a new climate state characterized by more frequent heatwaves, permafrost melting, and increased wildfires. Such changes could have widespread and serious consequences, emphasizing the need for adaptation strategies to mitigate these risks.

#### 3.1.3 Changing Patterns in Cold Waves across Kazakhstan

**Figure 5:**
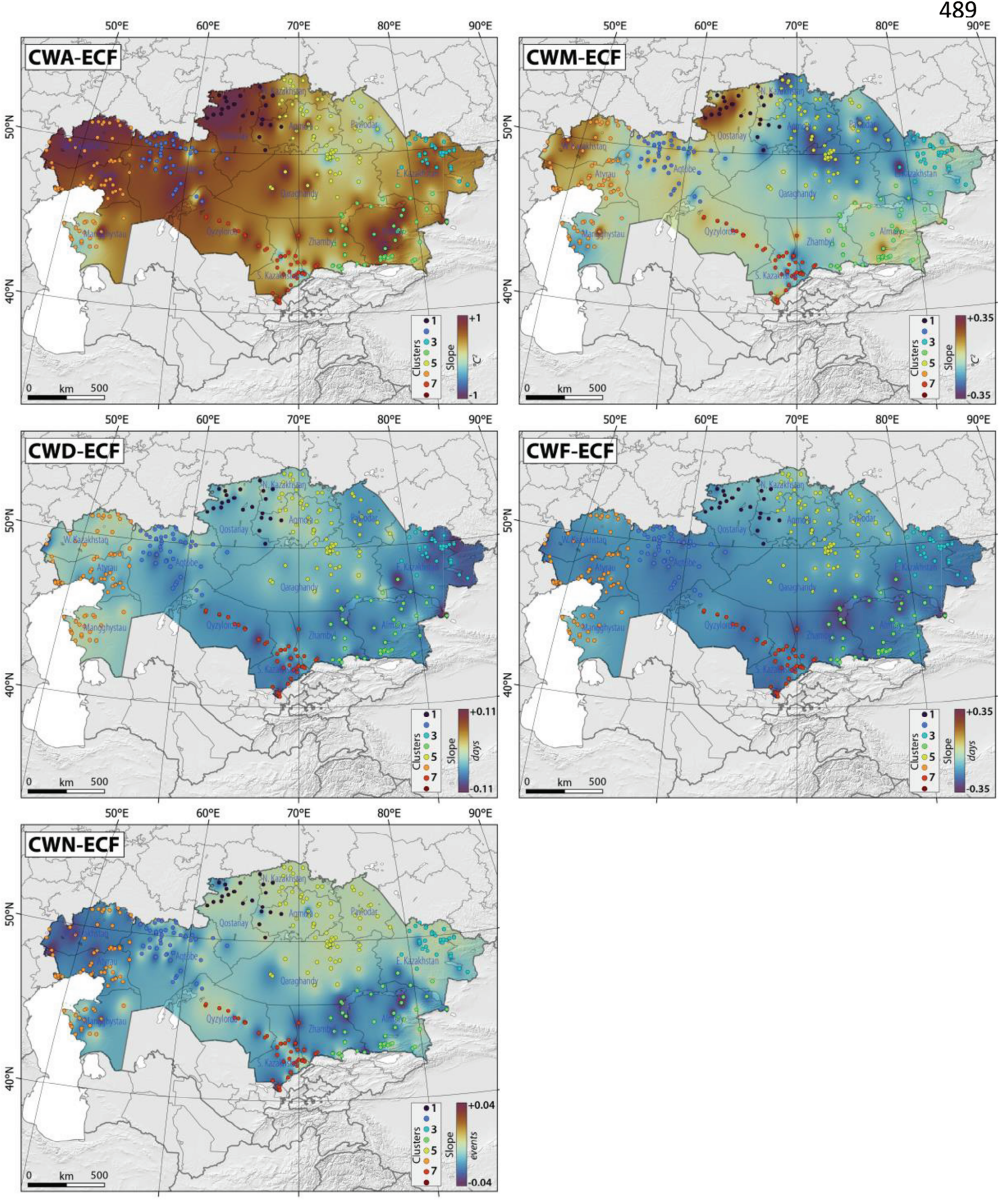
The slope of spatial‐temporal changes in the CWA‐ECF, CWM‐ECF, CWD‐ECF, CWF‐ECF, and CWN‐ECF in Kazakhstan, Central Asia, between 1959 and 2021. Please note that colored dots and color scale refer to identified clusters and slope of changes, respectively, in Kazakhstan.

**Figure** **5** shows a consistent decline in the spatiotemporal distribution of coldwave characteristics, including CWA, CWD, CWF, CWM, and CWN, all defined through the Excess Cold Factor, across Kazakhstan between 1959 and 2021. These changes were particularly pronounced in regions such as northwest Kazakhstan (excluding CWD, which remained relatively stable), east Kazakhstan (excluding CWN, which remained relatively stable), south Kazakhstan, and southeast Kazakhstan.

In contrast, CWA and CWM exhibited significant increases across extensive areas of Kazakhstan, indicating an escalation in the occurrence of cold waves characterized by the lowest minimum daily temperatures observed among all cold wave events. CWA displayed a marked increase across most of Kazakhstan, with exceptions in certain parts of West Kazakhstan and Northeast Kazakhstan (Figure 5A,C). Furthermore, the magnitude of cold waves notably rose in regions like north Kazakhstan and west Kazakhstan over the study period (Figure 5E).

Table S4, Supporting Information presents details of the most notable Sen's slope values, denoting the most prominent variations (increase and/or decrease) in CWA, CWD, CWF, CWM, and CWN as defined by the Excess Cold Factor across various clusters in Kazakhstan (Table S4, Supporting Information). Among these indices, Cluster 4 (Kapshagay, slope of 1.45) and Cluster 1 (Tobyl, slope of 0.324) demonstrated the most substantial significant increases in CWA (*p*‐value < 0.05) and CWM (*p*‐value > 0.05). Conversely, the most pronounced decreases in CWD, CWF, and CWN were observed in various cities within Cluster 4.

Recent research investigations have provided evidence indicating that the FD index, a metric used to measure the number of days with a minimum daily temperature below 0 °C, demonstrates a discernible pattern characterized by a significant decrease of 2–6 days over a period of 10 years in Kazakhstan.^[^ ^52^ ^]^ The observed decline in the FD index has a notable prominence within the geographically southern portions of the country, characterized by higher temperatures. In a similar vein, the ID index, denoting the count of days with a minimum daily temperature below 10 °C, exhibited a consistent pattern of decrease ranging from 2 to 5 days throughout Kazakhstan.^[^ ^5^, ^52^ ^]^ Salnikov et al.^[^ ^25^, ^52^ ^]^ observed a decline in the duration of cold waves over time. However, the spatial distribution of cold wave duration did not demonstrate a consistent pattern and instead exhibited a more dispersed arrangement over the studied region.

### 3.2 Changing Patterns in the Consecutive Dry Days in Kazakhstan

**Figure 6:**
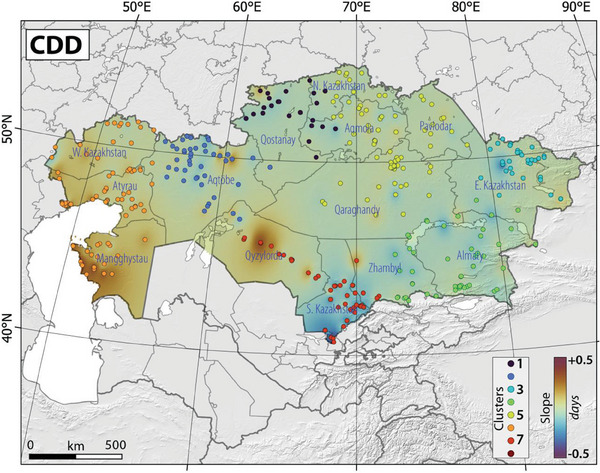
The slope of spatial‐temporal changes in the CCD in Kazakhstan, Central Asia, between 1959 and 2021. Please note that colored dots and color scale refer to identified clusters and slope of changes, respectively, in Kazakhstan.

**Figure** **6** illustrates a notable spike in the temporal and spatial distribution of CDD in west Kazakhstan and south Kazakhstan, with a slight increase observed in the remaining regions of Kazakhstan (Figure 6). Table S5, Supporting Information presents details of the most notable Sen's slope values, denoting the most prominent variations in CDD across various clusters in Kazakhstan (Table S5, Supporting Information). Table S5, Supporting Information provides information about the highest Sen's slope values, signifying the most substantial changes in CDD across different clusters in Kazakhstan (Table S5, Supporting Information). Notably, statistically significant increases were observed in Clusters 7, 6, and 2, with corresponding Sen's slope values of 0.486, 0.385, and 0.211, respectively.

The Maximum CDD demonstrated an anticipated pattern where its values increased from north to south of Kazakhstan, influenced by variations in terrain and notably affected by mountainous regions in southeast Kazakhstan. The index values ranged from 25 to 35 days in mountainous areas, gradually extending to 70 to 93 days in the southern parts. Notably high values of 74 to 93 days were observed in regions such as Turkestan, Kyzylorda, and the extreme southern area of Mangystau. The peak value was situated in the south, specifically in the Turkestan region.^[^ ^5^, ^52^ ^]^ Conversely, CWD increased from the southwest to the north, with particular emphasis on the northeast regions of the country. There is a distinct minimum around the Lake Balkhash area, while a maximum is concentrated in the Aktobe region.^[^ ^5^, ^52^ ^]^

**Figure 7:**
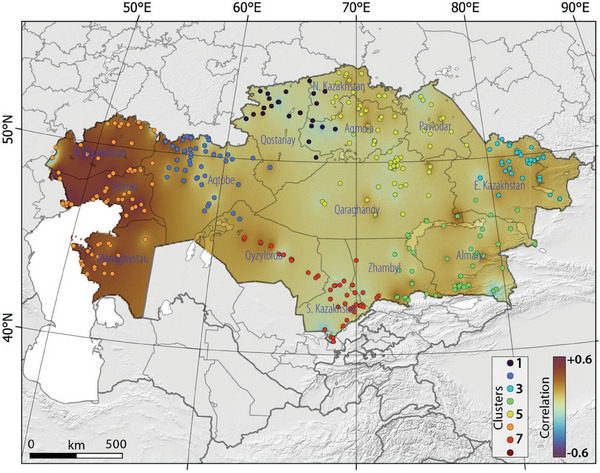
The correlation map between HWN‐ECF and CCD in Kazakhstan, Central Asia, between 1959 and 2021. Please note that colored dots and color scale refer to identified clusters and slope of changes, respectively, in Kazakhstan.

**Figure** **7** illustrates the correlation map between HWN‐ECF and CCD in Kazakhstan, Central Asia, between 1959 and 2021. The correlation maps between HWN, as defined by the Excess Heat Factor, TX90p, TN9p, and CDD depicted a substantial positive correlation between the incidence of heatwaves and periods of consecutive dry days in specific regions of Kazakhstan (Figure 7). It is important to note that correlation maps between CDD and HWN defined by TX90p and TN9p, are not provided. The correlation analysis revealed a stronger positive correlation in west Kazakhstan, with a relatively weaker positive correlation observed in other parts. Our results indicate a noteworthy alignment between heightened susceptibility to consecutive dry days and the occurrence of heatwaves, except for certain areas like south Kazakhstan, where the correlation displayed a negative trend. This negative correlation implies an increase in heatwave frequency alongside a decrease in the occurrence of consecutive dry days. Our clustering analyses demonstrated that the most robust and statistically significant correlation was identified in cluster 6 (Aqkol) and cluster 2 (Arkrab), yielding correlation coefficients of 0.61 (*p*‐value = 0.01) and 0.46 (*p*‐value = 0.00), respectively (Table S6, Supporting Information).

Hence, the conclusive evidence of a positive correlation between the co‐occurrence of heatwaves and consecutive dry days, as discovered in this study, substantially strengthens the case for considering the simultaneity of these events when evaluating the potential impacts of extreme weather in Kazakhstan. Gaining a more refined comprehension of such concurrent occurrences, their prevalence, and the underlying mechanisms governing this interdependence holds paramount importance in delivering more accurate forecasts and future projections of compound events within the context of a warming climate. This significance is particularly heightened for regions characterized by elevated risk levels, where such insights could be instrumental in comprehending future risks and shaping the strategic planning of adaptive measures. This holds especially true for adaptation strategies addressing heatwaves, which often involve elements like green spaces, water management, and air‐conditioning, assuming an absence of dry conditions.^[^ ^54^ ^]^

### 3.3 Health Risk Assessment Associated with Heat Waves

To bridge the discussion between the examinations of the spatio‐temporal distribution of extreme temperature events, focusing on heatwaves, the assessment of health impacts associated with heatwaves and their temporal distribution, is essential to explore the interplay between these climatic patterns and their consequences on human well‐being. The analysis of heatwaves variations over time and space lays the foundation for understanding how these fluctuations correlate with health outcomes, providing a comprehensive perspective on the complex relationship between climatic conditions and public health.

We examined the impact of heatwaves on specific health conditions within Kazakhstan. We selected a range of morbidities, including all cause and cardiovascular (CVD) mortalities, which we hypothesized, might be influenced by short‐term fluctuations in temperature. Tables S7–S12, Supporting Information show the number of all‐cause and CVD mortalities associated with different definitions of heatwave in Kazakhstan (Tables S7–S12, Supporting Information). **Table** **3** shows the Mann‐Kendall trend analysis for all‐cause and CVD mortalities associated with different definitions of heatwaves in Kazakhstan from 1959 to 2021. Our findings indicated a consistent rise in both all‐cause and CVD mortalities linked to various heatwave definitions (Table 3) across Kazakhstan. There was a statistically notable rise (*p*‐value < 0.05) in the number of all‐cause and CVD mortalities in Kazakhstan for various definitions of heatwaves. The most notable statistically significant increments (*p*‐value < 0.05) were witnessed in All‐cause mortality_HWN‐TX90_ and All‐cause mortality _HWN‐EHF_, showing changes with slopes of 3.30 and 3.12, respectively.

**Table 3:** The Mann‐Kendall trend analysis for all‐cause and CVD mortalities associated with different definitions of heatwaves in Kazakhstan from 1959 to 2021.

Variable	Sen's slope	*p*‐value (Two‐tailed)
All‐cause mortality _HWN‐TX90_	3.30	<0.0001
All‐cause mortality _HWN‐TN90_	0.62	<0.0001
All‐cause mortality _HWN‐EHF_	3.12	<0.0001
CVD mortality_HWN‐TX90_	1.40	0.022
CVD mortality _HWN‐TN90_	1.40	0.025
CVD mortality _HWN‐EHF_	1.40	0.01

Table S13, Supporting Information shows the Mann‐Kendall trend analysis for all‐cause and CVD mortalities associated with different definitions of heatwaves in Kazakhstan from 1959 to 2021. The number of all‐cause mortalities displayed a statistically significant increase (*p*‐value < 0.05) across all identified clusters in Kazakhstan, under different heatwave definitions (except clusters 1, 3, and 4 where changes in HWN‐TN90 were not observed). Additionally, statistically significant elevations in CVD mortality (*p*‐value < 0.05) were observed in clusters 6 and 7 across Kazakhstan, considering all definitions of heatwaves (Table S13, Supporting Information). Notably, despite the overall increase in heatwaves (across all definitions), cluster 5 exhibited a decline in cardiovascular (CVD) mortality, which could potentially be linked to demographic changes within this cluster (Table S13, Supporting Information). Cluster 7 demonstrated the most pronounced surges in both all‐cause and CVD mortalities, as indicated by Sen's slope values of 1.44, 0.25, 1.34, 0.88, 0.94, and 0.92 All‐cause mortality _HWN‐EHF_, All‐cause mortality _HWN‐TX90_, All‐cause mortality _HWN‐TN90_, CVD mortality _HWN‐EHF_, CVD mortality _HWN‐TN90_, CVD mortality_HWN‐TX90,_ respectively.

**Table** **4** illustrates the health outcomes associated with various definitions of heatwaves, along with the annual count of heatwaves in Kazakhstan during the study period. It is important to highlight that the distribution of locations and their demographic data is not uniform across different clusters. To evaluate the impact of heatwaves on mortalities in Kazakhstan, our results captured different aspects of heatwaves and their associated health outcomes. Across all clusters, HWN‐TX90 is consistently associated with the highest average number of heatwaves (4426) and related all‐cause mortalities (1242), indicating that heatwaves defined by maximum temperature thresholds have the greatest impact. In contrast, HWN‐TN90 shows a similar average number of heatwaves (4424) but results in significantly lower all‐cause mortalities (229). Meanwhile, HWN‐EHF presents the fewest average heatwaves (4000), yet still results in substantial average all‐cause mortalities (1129), suggesting that it may capture more severe but less frequent events.

**Table 4:** Associated health outcomes for different definitions of heatwaves and the number of annual heatwaves in Kazakhstan during the study period.

*All‐cause mortalities are calculated from 1959 to 2021*.							
*Variable*	*Cluster1*	*Cluster 2*	*Cluster 3*	*Cluster 4*	*Cluster 5*	*Cluster 6*	*Cluster 7*
HWN‐TX90	2409	2016	2907	5237	6743	5242	6427
All‐cause mortality_HWN‐TX90_	563	632	550	643	2079	1322	2904
HWN‐TN90	2395	2003	2943	5306	6715	5200	6405
All‐cause mortality_HWN‐TN90_	85	119	103	58	435	253	553
HWN‐EHF	2131	1790	2624	4854	5992	4675	5938
All‐cause mortality _HWN‐EHF_	472	554	507	602	1865	1162	2742

*CVD mortalities are calculated from 1990 to 2019*.							
*Variable*	*Cluster1*	*Cluster 2*	*Cluster 3*	*Cluster 4*	*Cluster 5*	*Cluster 6*	*Cluster 7*
HWN‐TX90	1240	1112	1532	2853	3323	2969	4129
CVD mortality_HWN‐TX90_	94	113	90	66	365	238	628
HWN‐TN90	1242	1122	1555	2906	3314	2966	4140
CVD mortality_HWN‐TN90_	95	114	89	65	364	241	641
HWN‐EHF	1092	1023	1395	2570	2982	2649	3890
CVD mortality _HWN‐EHF_	76	99	86	56	321	215	594

Regarding cardiovascular disease (CVD) mortalities, HWN‐TX90 again shows the highest averages, with 2451 heatwaves and 228 CVD mortalities. HWN‐TN90 records a slightly higher average number of heatwaves (2464) but similar CVD mortalities (230), while HWN‐EHF has the fewest average heatwaves (2229) and related CVD mortalities (207). These findings consistently demonstrate that heatwaves defined by maximum temperature thresholds (HWN‐TX90) lead to the most severe health outcomes, both for all‐cause and CVD mortalities. Clusters 5 and 7 report the highest number of heatwaves and associated mortalities across all definitions, reflecting their increased vulnerability to intense heatwaves. For instance, Cluster 7 shows the maximum number of HWN‐TX90 heatwaves (6427) and all‐cause mortalities (2904), along with 4129 HWN‐TX90 heatwaves and 628 CVD mortalities. Similarly, Cluster 5 ranks among the highest for heatwaves and mortalities across definitions. On the other hand, Clusters 1 and 2 consistently show the lowest heatwave counts and related mortalities, indicating a lower risk. It is noteworthy that all‐cause mortalities were calculated from 1959 to 2021, while CVD mortalities were assessed from 1990 to 2021 due to data availability. This difference in timeframes aligns with observed variations in mortality estimates across clusters, where Clusters 5 and 7 show the highest mortality counts, while Clusters 1 and 2 exhibit the lowest (Table 4). The comparison underscores that the choice of heatwave definition significantly influences the observed health impacts, with maximum temperature thresholds demonstrating the most severe outcomes, especially in regions experiencing frequent and intense heatwaves. Understanding these differences is crucial for effective public health planning and targeted climate adaptation strategies in Kazakhstan. By considering regional variations and the specific characteristics of each heatwave definition, this analysis supports the development of interventions aimed at protecting vulnerable populations across the country.

There is a consensus that the augmented health impacts on heatwave days exhibit variability across studies utilizing distinct heatwave definitions.^[^ ^55^, ^56^, ^57^, ^58^ ^]^ Our findings of heat‐related morbidities during heatwaves are consistent with prior research.^[^ ^2^, ^42^, ^59^, ^60^, ^61^ ^]^ For instance, during the 2006 heatwaves in California, there was a tenfold increase in hospitalizations caused by heat‐related sicknesses.^[^ ^62^ ^]^ Heatwaves exhibited an elevated occurrence of heat‐related morbidities such as dehydration and heat stroke.^[^ ^3^ ^]^ A recently conducted study in China showed that heatwaves elevated the risk of total non‐accidental mortality by 15.7% (95% CI: 12.5, 18.9) in comparison to periods without heatwaves. Additionally, the risk of cardiovascular‐related mortality increased by 22.0% (95% CI: 16.9, 27.4).^[^ ^57^ ^]^ Another study involving 43 U.S. communities found a 2.49% rise in heatwave‐associated mortality risk for each 1°F increase in heatwave intensity, as well as a 0.38% increase for each additional day in heatwave duration. The mortality rate jumped by 5.04% (95% CI: 3.06, 7.06) during the first summer heatwave and by 2.65% (95% CI: 1.14, 4.18) during subsequent heatwaves, compared to non‐heatwave days. Health risks associated with cardiovascular diseases are especially vulnerable to increased temperatures.^[^ ^63^, ^64^, ^65^, ^66^ ^]^

Although extreme heat events have transitioned into regular occurrences during summer seasons across the world, leading to a notable rise in excess fatalities, many of the excess deaths and heat‐related health risks can be averted through effective heat action plans that integrate behavioral strategies and biophysical solutions. As climate change continues, the likelihood of heightened heat‐related mortality is projected to escalate, particularly with more pronounced risks tied to greater degrees of global warming. So, urgent and synchronized efforts are imperative to heighten public consciousness about temperature's implications for health, implement effective heatwave early warning systems, and establish enhanced health safeguarding approaches to combat the impacts of global warming. The divergent mortality impacts of suboptimal temperatures across different regions and localities underline the importance of thorough substantial investments in research and risk management measures and investigation to formulate adaptive strategies that safeguard health against excessive heat.

Even though extreme heat events have evolved into recurring phenomena during summer seasons globally, resulting in a significant increase in excess fatalities, many of these deaths and associated heat‐related health risks can be mitigated through efficient heat action plans incorporating both behavioral strategies and biophysical solutions. As climate change persists, the potential for amplified heat‐related mortality is anticipated to escalate, especially with heightened risks associated with more substantial degrees of global warming. Thus, there is an urgent need for comprehensive and coordinated efforts to enhance public awareness about the health implications of temperature, implement effective heatwave early warning systems, and establish advanced health protection strategies to counteract the impacts of global warming. The varying impacts of suboptimal temperatures on mortality across diverse regions and localities underscore the importance of substantial investments in research and risk management, as well as thorough investigation, to formulate adaptive measures that effectively safeguard public health against the challenges of excessive heat.

### 3.4 Changes in Energy Demands and CO_2_ Emissions in Energy Production in Kazakhstan

To establish a connection between the examination of the spatio‐temporal distribution of heat/cold waves and the assessment of the energy demand and carbon footprint associated with these phenomena, it is crucial to investigate the reciprocal relationship between climatic patterns and energy consumption. Analyzing how heat/cold waves manifest over different regions and time periods provides insights into their impact on energy demand. Simultaneously, assessing the carbon footprint associated with these climatic events reveals the environmental implications of the energy requirements, forming a comprehensive understanding of the interconnected dynamics between climate, energy usage, and carbon emissions.

**Figure 8:**
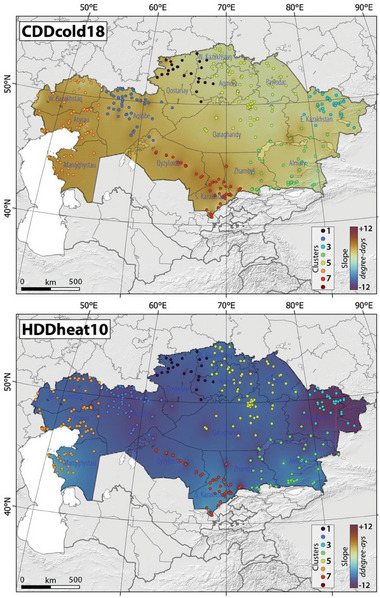
The slope of spatial‐temporal changes in the CDD18 and HDD10 in Kazakhstan, Central Asia, between 1959 and 2021. Please note that colored dots and color scale refer to identified clusters and slope of changes, respectively, in Kazakhstan.

**Figure** **8** shows the slope of spatial‐temporal changes in the CDD18 and HDD10 in Kazakhstan, Central Asia, between 1959 and 2021. Our results indicated a notable increase in the spatial‐temporal distribution of CDD18 and HDD10 in regions such as northwest Kazakhstan, west Kazakhstan, and south Kazakhstan, accompanied by a minor increase in other parts of Kazakhstan (Figure 8A). Conversely, the application of the Mann‐Kendall trend analysis method reveals a significant decrease in heating‐degree days in east Kazakhstan, west Kazakhstan, and northwest Kazakhstan, with a marginal decline detected in other regions of Kazakhstan (Figure 8).

Table S14, Supporting Information presents details of the most notable Sen's slope values, denoting the most prominent variations in CDD18 and HDD10, signifying the most substantial changes in cooling and heating‐degree days across different clusters in Kazakhstan. Significantly, there were notable increases in CDD18 observed in Cluster 7, Cluster 4, and Cluster 6, with corresponding Sen's slope values of 6.043, 5.602, and 5.362, respectively. Conversely, the smallest change was recorded in Cluster 3, where the Sen's slope was 0.142 (*p*‐value < 0.05), indicating a minor increase. On the other hand, when it comes to HDD10, there were statistically significant decreases observed in Cluster 3, Cluster 7, and Cluster 2, with corresponding Sen's slope values of −12.462, −10.949, and −10.521, respectively. In contrast, the least change in HDD10 was observed in Cluster 6, with a Sen's slope of −3.047 (*p*‐value < 0.05).

**Table** **5** presents the energy demand analysis for both heating and cooling requirements in various identified clusters across Kazakhstan from 1959 to 2021, based on the current energy generation system configuration. Despite the general decrease in the number of coldwaves and their associated indices and the increase in the number of heatwaves and their related indices, our analysis indicates that heating‐degree days still exceed cooling‐degree days in various clusters across Kazakhstan (Figures 4, 5, and 8). Cluster 3 exhibited the highest heating‐degree days, with an average value of 3528, while Cluster 7 had the lowest, with 1641‐degree days. Conversely, the highest cooling‐degree days were recorded in Cluster 7, with 889‐degree days, whereas Cluster 1 displayed the lowest average cooling‐degree days among the identified clusters in Kazakhstan during the same period (Table 5). These changes in average cooling‐degree days correspond with our heatwave analysis, where Cluster 7 exhibited a higher propensity for increased intensity, frequency, and number of heatwaves compared to Cluster 1. Furthermore, Cluster 3 displayed a relatively higher tendency for more intense and frequent cold waves, while Cluster 7 was more susceptible to more intense but less frequent cold waves in Kazakhstan (Table 5).

**Table 5:** The energy demand (kWh) analysis and associated CO_2_ emissions (kg kWh^−1^) for heating and cooling purposes in different clusters in Kazakhstan between 1959 and 2021.

*Current energy generation system*								
Cluster	HDD10 (degree days)	CDD18 (degree days)	Energy generation for heating	Energy generation for cooling	Total Energy generation	CO_2_ emission for heating	CO_2_ emission for cooling	Total CO_2_ emission
1	3450	271	82.81	6.51	89.32	69.58	5.47	75.05
2	3018	497	72.43	11.92	84.35	60.86	10.01	70.88
3	3528	229	84.67	5.50	90.17	71.14	4.62	75.77
4	2421	495	58.09	11.87	69.97	48.81	9.98	58.79
5	3460	264	83.04	6.34	89.38	69.78	5.32	75.10
6	2035	807	48.85	19.36	68.21	41.04	16.27	57.31
7	1641	889	39.38	21.32	60.70	33.09	17.92	51.00

Based on our analysis of the variations in heating and cooling‐degree days in Kazakhstan over the study period, it is evident that energy generation for heating purposes exceeded that for cooling purposes in all identified clusters (Table 5). Figure S3, Supporting Information shows the temporal and spatial distribution of energy generation for both heating and cooling needs in Kazakhstan during the study period. Notably, our findings indicate that clusters with higher energy generation for heating purposes corresponded to lower energy generation requirements for cooling needs. For instance, Cluster 3 demonstrated the highest total energy demand at 90.17 kWh, driven primarily by heating requirements (84.67 kWh), corresponding to its high HDD10 value. In contrast, Cluster 7 had the lowest total energy demand at 60.70 kWh, characterized by lower heating demand (39.38 kWh) but higher cooling demand (21.32 kWh), consistent with its higher CDD18 value. Cluster 1 reported a total energy demand of 89.32 kWh, largely dominated by heating (82.81 kWh), while Cluster 6 showed a more balanced energy demand of 68.21 kWh, with 48.85 kWh attributed to heating and 19.36 kWh to cooling. The average total energy generation for both heating and cooling purposes, irrespective of building type, was 552 kWh across Kazakhstan, summed over the period from 1959 to 2021, with an average yearly energy generation of ≈8.76 kWh. This variation aligns with the findings that clusters with higher heating demands corresponded to lower energy requirements for cooling. It is important to note that building heat loss rates can vary due to evolving building efficiency standards and changing household expectations for thermal comfort. Therefore, our approach involves calculating energy demands per unit of heat loss for heating and thermal transmittance per square meter for cooling, independently of building types and efficiency standards.

**Figure 9:**
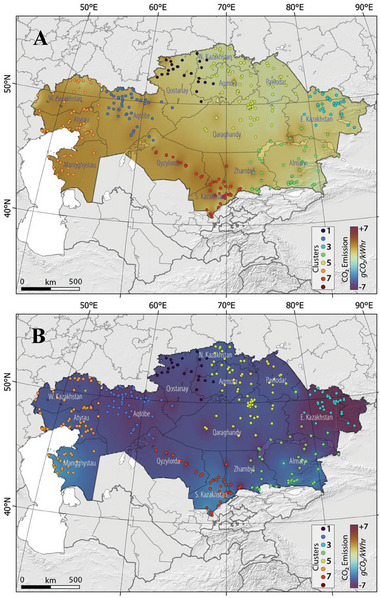
The trend of changes in A) CO_2_ emissions per unit of energy generation for cooling (g CO_2_/kWh) and B) CO_2_ emissions per unit of energy generation for heating (g CO_2_/kWh) in Kazakhstan, Central Asia, between 1959 and 2021. Please note that colored dots and color scale refer to identified clusters and slope of changes, respectively, in Kazakhstan.

Because it is essential to estimate power plant CO_2_ emissions to evaluate their environmental impact, monitor their progress toward emissions reduction goals, and guide policy, we decided to estimate the quantity of CO_2_ emissions produced per unit of energy generation, considering the configuration of the energy generation in Kazakhstan (Figure 3 and Table 1). **Figure** **9** shows the trend of changes in CO_2_ emissions per unit of energy generation for cooling and purposes in Kazakhstan, Central Asia, between 1959 and 2021. Table 5 shows the energy demand analysis and associated CO_2_ emissions for heating and cooling purposes in different clusters in Kazakhstan between 1959 and 2021. CO_2_ emissions from heating were highest in Cluster 3, totaling 71.14 kg kWh^−1^, while the lowest emissions were recorded for cooling purposes in the same cluster, at 4.62 kg kWh^−1^ (Table 5 and Figure 9). In contrast, Cluster 7 had the lowest total CO_2_ emissions at 51.00 kg kWh^−1^, with a relatively higher share from cooling (17.92 kg kWh^−1^) compared to heating (33.09 kg kWh^−1^). These differences reflect the energy demand patterns across clusters, with clusters like 3, 1, and 5 exhibiting over 90 percent of total CO_2_ emissions from heating, while clusters like 7 show a greater proportion of emissions from cooling. The current power system configuration in Kazakhstan produced total mean CO_2_ emissions of 464 kg kWh^−1^ for both heating and cooling purposes, aligning with spatial and temporal distribution trends illustrated in Figure 8.

**Figure 10:**
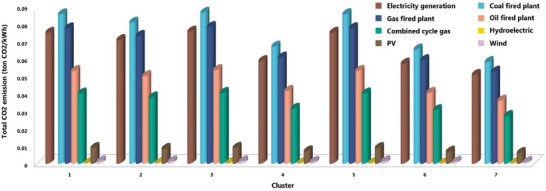
Total estimated CO_2_ emission (ton CO_2_/kWh) from different proposed fossil fuel types and renewable sources in different identified clusters in Kazakhstan, Central Asia, between 1959 and 2021.

Additionally, we also calculated the CO_2_ emissions for various potential alternatives involving different fuel sources, encompassing coal, natural gas, oil, combined‐cycle gas, and renewable options such as hydroelectric, photovoltaics (PV), and wind (**Figure** **10** and Table S15, Supporting Information). Figure 10 shows total estimated CO_2_ emission from different proposed fossil fuel types and renewable sources in different identified clusters in Kazakhstan, Central Asia, between 1959 and 2021. Moreover, Table S15, Supporting Information shows the energy demand analysis and associated CO_2_ emissions for both heating and cooling purposes in different clusters in Kazakhstan between 1959 and 2021 for various potential alternatives involving different fuel sources, encompassing coal, natural gas, oil, combined‐cycle gas, and renewable options such as hydroelectric, photovoltaics (PV), and wind energy.

Among fossil fuel alternatives, combined‐cycle gas exhibited the lowest emissions, totaling 248.50 kg kWh^−1^, with 15% allocated for cooling and 85% for heating. Conversely, among the proposed renewable options, photovoltaics had the highest emissions, amounting to 55.20 kg kWh^−1^, while hydroelectric power demonstrated the lowest emissions at 2.21 kg kWh^−1^. Notably, the results indicated that the current energy generation system in Kazakhstan resulted in lower CO_2_ emissions when compared to full coal‐fired and gas power plants but exhibited higher emissions in comparison to other available alternatives (Figure 10 and Table S15, Supporting Information).

These findings underscore the urgent need to transition from fossil fuels as the primary energy source due to their significant environmental impacts. Fossil fuels as the primary source of national energy pose significant threats to the environment due to the emission of CO_2_ gas during combustion. This contributes to the accumulation of greenhouse gases and an increase in the carbon footprint. Energy consumption is directly linked to climate change, leading to higher greenhouse gas emissions and its associated consequences.^[^ ^1^, ^67^, ^68^ ^]^ To achieve the goal of limiting global warming to below 1.5 °C, as specified in the Paris Agreement, it is crucial to cut annual greenhouse gas emissions by half in the coming years. Furthermore, adhering to net‐zero emissions commitments could assist in meeting the Paris Agreement's objective of keeping warming well below 2 °C.^[^ ^1^, ^67^, ^68^, ^69^ ^]^ The analysis of potential energy alternatives in this study, including renewable sources like hydroelectric and photovoltaics, highlights viable pathways for emission reduction.

To effectively address climate change and protect public health and air quality, a comprehensive set of measures is required. This includes the development of environmentally friendly regulations, enhancement of energy efficiency, adherence to international reporting standards, and increased public awareness of the environmental impacts of energy production.^[^ ^69^, ^70^ ^]^

A primary strategy for sustainable development is to prioritize the reduction of carbon emissions by minimizing dependence on fossil fuels. The transition to renewable energy sources not only advances sustainability objectives but also prevents additional carbon emissions, thereby mitigating adverse environmental impacts at the source. Transitioning energy systems must also address the growing challenges of urban heat islands and heatwaves. Efforts must also focus on addressing the challenges of both urban heat islands and cold spells, necessitating the implementation of climate‐resilient infrastructure and the development of effective urban climate management plans. These plans integrate measures for resilience to both heat and cold, combining mitigation strategies such as green and blue infrastructure and reflective surfaces for cooling, with adaptation strategies like enhanced insulation, efficient heating systems, and improved building codes to handle cold temperatures.^[^ ^71^, ^72^ ^]^

To facilitate the transition to renewable energy while ensuring urban heat resilience, it is essential to (1) implement new regulations and policies that promote the widespread adoption of renewable energy sources while supporting infrastructure development that can handle both heat and cold extremes in urban and rural areas, (2) continue technological advancements to enhance the efficiency and cost‐effectiveness of renewable energy processes, incorporating innovations like cool pavements and reflective building materials to reduce urban heat, as well as high‐efficiency insulation materials to protect against cold, (3) develop efficient carbon capture methods to mitigate climate change and contribute to more resilient urban environment, (4) Produce highly efficient renewable energy harvesting devices to improve overall efficiency and reduce costs, and (5) expand the use of fuel cells in the transportation sector to further reduce harmful gas emissions and protect the environment and help manage temperature extremes by reducing reliance on conventional heating and cooling systems.^[^ ^72^, ^73^, ^74^, ^75^ ^]^

Achieving a sustainable, low‐carbon energy system while developing resilient urban environments requires cross‐sectoral integration across energy, transportation, urban development, and public health. These strategies align with broader Sustainable Development Goals, promoting sustainable urban growth and effective climate adaptation for both cold and heat challenges.^[^ ^72^ ^]^

## 4 Summary and Conclusion

Here, we investigated the adverse effects of heatwaves on human well‐being and energy utilization, breaking new ground in our understanding of these critical issues. Our findings were drawn from extensive datasets (ECMWF Reanalysis v5), comprising extreme air temperature and precipitation, covering a span from 1959 to 2021, we followed the guidelines established by the WMO. Employing spatial and temporal evaluations of specialized climatic indices, we systematically analyzed associated trends. By leveraging the K‐means clustering method, we segmented Kazakhstan into seven distinct clusters.

Our findings unveiled significant shifts in temperature patterns. Notably, West Kazakhstan, Northwest Kazakhstan, and East Kazakhstan experienced marked reductions in the percentage of cool nights and days (*p*‐value < 0.05). In contrast, warm nights and days exhibited pronounced increases in these regions, along with South Kazakhstan (*p*‐value < 0.05).

The most striking heatwave‐related trends were observed in the western and southwestern regions, marked by a substantial increase in intensity (slope of 0.40, *p*‐value < 0.05), frequency (slope of 0.55, *p*‐value < 0.05), and overall occurrence (slope of 0.09, *p*‐value < 0.05). On the other hand, despite heightened cold wave intensity across the country, we observed a decline in both their frequency (slope of −0.11, *p*‐value < 0.05) and occurrence (slope of −0.04, *p*‐value < 0.05) over time.

Furthermore, we found a noteworthy surge in CDD occurrences in West Kazakhstan and South Kazakhstan (*p*‐value < 0.05), with slight increases in other regions from 1959 to 2021. Interestingly, our correlation maps highlighted a robust positive association between heatwave incidence and prolonged periods of dry days in specific areas of Kazakhstan (*p*‐value <0.05), affirming heightened susceptibility during such conditions. Nevertheless, South Kazakhstan exhibited an exception, displaying a negative correlation trend.

Despite the provided insights by our study into the health impacts of extreme temperatures in Kazakhstan, there are several limitations that must be addressed.

First, the lack of daily mortality data in the region necessitated the use of a simplified estimation method, wherein annual mortality totals were divided by the number of days in a year (365 or 366). Although this approach is commonly employed in data‐scarce settings, it does not capture daily fluctuations in mortality, especially during extreme temperature events, potentially leading to both overestimations and underestimations.^[^ ^43^, ^44^ ^]^ Second, the study utilizes global meta‐analyses to estimate relative risks (RR) for heat‐/coldwaves. While this approach offers comprehensive and statistically sound estimates, it assumes that relative risks derived from diverse global contexts apply directly to the specific climatic, demographic, and socioeconomic conditions of Kazakhstan.^[^ ^43^, ^44^ ^]^ This assumption introduces potential bias by not accounting for local variations in vulnerability and adaptive capacity.

Third, the lack of region‐specific studies hindered the ability to conduct a comprehensive health risk assessment for coldwaves, as no global meta‐analyses or localized data were available. Consequently, the findings regarding coldwave impacts remain incomplete and call for further investigation. Finally, the study's scope was confined to heat‐/coldwaves, excluding warm and cold spells, which may also contribute significantly to health risks. As a result, the analysis does not encompass the full spectrum of temperature‐related health impacts. These limitations emphasize the need for more region‐specific data collection and epidemiological studies, which would enhance the accuracy and applicability of future health risk assessments in Kazakhstan and Central Asia.

Shifting our focus to public health, our study investigated the impact of heatwaves on various health conditions, revealing consistent and statistically notable spikes in all‐cause (*p*‐value < 0.0001) and CVD (*p*‐value < 0.05) mortalities across Kazakhstan, under various heatwave definitions. As there is no worldwide assessment available for the repercussions of cold waves, their health effects were not evaluated. However, we found a pronounced surge in cooling‐degree days and a reduction in heating‐degree days. Irrespective of building type, the average total energy consumption stood at 552 kWh across the country summed over the period from 1959 to 2021, with an average yearly energy generation of ≈8.76 kWh. Assessing the associated carbon footprint stemming from energy generation, primarily coal power plants, our analysis estimated mean CO_2_ emissions at 464 kg per kWh for both heating and cooling purposes in Kazakhstan during the same period.

Considering the anticipated rise in heatwave frequency and duration due to climate change, we emphasize the critical need for comprehensive health planning to mitigate their adverse effects, particularly among vulnerable populations. Additionally, our findings highlight the urgency of transitioning from fossil fuels to green energy sources to curb carbon emissions and minimize environmental impact.

## Conflict of Interest

The authors declare no conflict of interest.

## Supporting information



Supporting Information

Supporting Information

Supporting Information

## Data Availability

The data that support the findings of this study are available from the corresponding author upon reasonable request.
