# Supplementary material for: Extreme Temperature Events in Kazakhstan and Their Impacts on Public Health and Energy Demand
Source: Glob Chall. 2024 Dec 19;9(2):2400207. doi: 10.1002/gch2.202400207 (PMC11802326; doi:10.1002/gch2.202400207)
Supplement: Supplementary file 1 — Supporting Information [file GCH2-9-2400207-s001.docx]

**Extreme Temperature Events in Kazakhstan and their Impacts on Public Health and Energy Demand**

Parya Broomandi^1,2,3#^, Alfrendo Satyanaga^1^, Mehdi Bagheri^2*^, Mostafa Hadei^4,5^, David Galán-Madruga^6^, Ali Mozhdehi Fard^7^, Adib Roshani^7^, Aram Fathian^3,8,9^, Amir Nourian^10^, Michael Leuchner^11^, Klaus Reicherter^3^, Mehdi Hamidi^7,12^, Prashant Kumar^13,14*^, Jong Ryeol Kim^1*^

^1^ Department of Civil and Environmental Engineering, School of Engineering and Digital Sciences, Nazarbayev University, Kabanbay Batyr Ave. 53, Astana 010000 Kazakhstan.

^2^Department of Electrical and Computer Engineering, School of Engineering and Digital Sciences, Nazarbayev University, Kabanbay Batyr Ave. 53, Astana 010000 Kazakhstan.

^3^Neotectonics and Natural Hazards Institute, RWTH Aachen University, Aachen 52056, Germany.

^4^Department of Health in Emergencies and Disasters, Tehran University of Medical Sciences, Tehran 1417613151, Iran.

^5^Climate Change and Health Research Center (CCHRC), Institute for Environmental Research (IER), Tehran University of Medical Sciences, Tehran 1439813118, Iran

^6^Department of Atmospheric Pollution, National Centre for Environment Health, Health Institute Carlos III. Ctra. Majadahonda a Pozuelo km 2.2, 28220 Madrid, Spain.

^7^Faculty of Civil Engineering, Babol Noshirvani University of Technology, Babol 484, Iran.

^8^UNESCO Chair on Coastal Geo-Hazard Analysis, Research Institute for Earth Sciences, Tehran 13185-1494, Iran.

^9^Water, Sediment, Hazards, and Earth-surface Dynamics (waterSHED) Lab, Department of Geoscience, University of Calgary, Calgary Alberta T2N 1N4, Canada.

^10^School of Science Engineering and Environment (SEE), University of Salford, Salford M5 4WT, Manchester, United Kingdom.

^11^Physical Geography and Climatology, Department of Geography, RWTH Aachen University, Wüllnerstr. 5b, 52062 Aachen, Germany.

^12^Institute for Geophysics and Meteorology, University of Cologne, Pohligstr.3, 50969, Cologne, Germany.

^13^Global Centre for Clean Air Research (GCARE), School of Sustainability, Civil and Environmental Engineering, Faculty of Engineering and Physical Sciences, University of Surrey, Guildford GU2 7XH, Surrey, United Kingdom.

^14^Institute for Sustainability, University of Surrey, Guildford GU2 7XH, Surrey, United Kingdom.

* Corresponding authors. Email: [jong.kim@nu.edu.kz](mailto:jong.kim@nu.edu.kz), and Phone: +7 (7172) 70-91-36.

Email: p.kumar@surrey.ac.uk, and Phone: +44 1483 68 2762.

Email: mehdi.bagheri@nu.edu.kz, and Phone: +7 (7172) 70-92-51

| **Table S1.** Climate indexes with their definitions and units used in current study. | | | | |
| --- | --- | --- | --- | --- |
| **ID** | **Index Name** | **Definition** | **Unit** | **Sectors of Economics** |
| CWD-ECF | Cold wave duration as defined by the Excess Cold Factor. | The length of the longest ‘cold wave’ identified by ECF_HWN. | days | Health, Agriculture and Food Security, Water Resources and Hydrology |
| CWA-ECF | Cold wave amplitude as defined by the Excess Cold Factor. | The minimum daily value in the coldest ‘cold wave’ (defined as the cold wave with lowest ECF_HWM). | °C^2^ | Health, Agriculture and Food Security, Water Resources and Hydrology |
| CWF-ECF | Cold wave frequency as defined by the Excess Cold Factor. | The number of days that contribute to ‘cold waves’ as identified by ECF_HWN. | days | Health, Agriculture and Food Security, Water Resources and Hydrology |
| CWN-ECF | Cold wave number as defined by the Excess Cold Factor. | The number of individual ‘cold waves’ that occur each year. | events | Health, Agriculture and Food Security, Water Resources and Hydrology |
| CWM-ECF | Cold wave magnitude as defined by the Excess Cold Factor. | The mean temperature of all ‘cold waves’ identified by ECF_HWN. | °C^2^ | Health, Agriculture and Food Security, Water Resources and Hydrology |
| HWD-EHF | Heatwave duration as defined by the Excess Heat Factor. | The length of the longest heatwave identified by HWN. | days | Health, Agriculture and Food Security, Water Resources and Hydrology |
| HWA-EHF | Heatwave amplitude as defined by the Excess Heat Factor. | The peak daily value in the hottest heatwave (defined as the heatwave with highest HWM). | °C^2^ | Health, Agriculture and Food Security, Water Resources and Hydrology |
| HWF-EHF | Heatwave frequency as defined by the Excess Heat Factor. | The number of days that contribute to heatwaves as identified by HWN. | days | Health, Agriculture and Food Security, Water Resources and Hydrology |
| HWN-EHF | Heatwave number as defined by the Excess Heat Factor. | The number of individual heatwaves that occur each summer (Nov – Mar in southern hemisphere and May – Sep in northern hemisphere). | events | Health, Agriculture and Food Security, Water Resources and Hydrology |
| HWM-EHF | Heatwave magnitude as defined by the Excess Heat Factor. | The mean temperature of all heatwaves identified by HWN. | °C^2^ | Health, Agriculture and Food Security, Water Resources and Hydrology |
| HWD-Tn90 | Heatwave duration as defined by the 90^th^ percentile of TN. | The length of the longest heatwave identified by HWN. | days | Health, Agriculture and Food Security, Water Resources and Hydrology |
| HWA-Tn90 | Heatwave amplitude as defined by the 90^th^ percentile of TN. | The peak daily value in the hottest heatwave (defined as the heatwave with highest HWM). | °C | Health, Agriculture and Food Security, Water Resources and Hydrology |
| HWF-Tn90 | Heatwave frequency as defined by the 90^th^ percentile of TN. | The number of days that contribute to heatwaves as identified by HWN. | days | Health, Agriculture and Food Security, Water Resources and Hydrology |
| HWN-Tn90 | Heatwave number as defined by the 90^th^ percentile of TN. | The number of individual heatwaves that occur each summer (Nov – Mar in southern hemisphere and May – Sep in northern hemisphere). | events | Health, Agriculture and Food Security, Water Resources and Hydrology |
| HWM-Tn90 | Heatwave magnitude as defined by the 90^th^ percentile of TN. | The mean temperature of all heatwaves identified by HWN. | °C | Health, Agriculture and Food Security, Water Resources and Hydrology |
| HWD-Tx90 | Heatwave duration as defined by 90^th^ percentile of TX. | The length of the longest heatwave identified by HWN. | days | Health, Agriculture and Food Security, Water Resources and Hydrology |
| HWA-Tx90 | Heatwave amplitude as defined by the 90^th^ percentile of TX. | The peak daily value in the hottest heatwave (defined as the heatwave with highest HWM). | °C | Health, Agriculture and Food Security, Water Resources and Hydrology |
| HWF-Tx90 | Heatwave frequency as defined by the 90^th^ percentile of TX. | The number of days that contribute to heatwaves as identified by HWN. | days | Health, Agriculture and Food Security, Water Resources and Hydrology |
| HWN-Tx90 | Heatwave number as defined by 90^th^ percentile of TX. | The number of individual heatwaves that occur each summer (Nov – Mar in southern hemisphere and May – Sep in northern hemisphere). | events | Health, Agriculture and Food Security, Water Resources and Hydrology |
| HWM-Tx90 | Heatwave magnitude as defined by the 90^th^ percentile of TX. | The mean temperature of all heatwaves identified by HWN. | °C | Health, Agriculture and Food Security, Water Resources and Hydrology |
| CDDcold18 | Cooling Degree Days. | Annual sum of TM - *n* (where *n* is a user-defined location-specific base temperature and TM > *n*). | degree-days | Health |
| HDDheat10 | Heating Degree Days. | Annual sum of TM - *n* (where *n* is a user-defined location-specific base temperature and TM <*n*). | degree-days | Health |
| TX10p | Amount of cool days. | Percentage of days when TX < 10th percentile. | % | Energy |
| TX90p | Amount of hot days. | Percentage of days when TX > 90th percentile. | % | Energy |
| TN10p | Amount of cold nights. | Percentage of days when TN < 10th percentile. | % | Energy |
| TN90p | Amount of warm nights. | Percentage of days when TN > 90th percentile. | % | Energy |
| CDD | Consecutive Dry Days. | Maximum number of consecutive dry days (when PR < 1.0 mm) | days | Health, Agriculture and Food Security, Water Resources and Hydrology |

### Heat-and coldwaves indices

### Three distinct heatwave definitions were employed using T_max_ (maximum temperature), T_min_ (minimum temperature), and EHF (excess heat factor), as explained below:

### CTX90pct: The threshold is the 90th percentile of T_max_ (maximum temperature) for each calendar day, considering a 15-day window. This means there is a unique percentile value for each day of the year, accounting for seasonal variations, with the window centered on the respective day. The use of a moving window ensures temporal dependence is considered while obtaining a sufficient sample size to determine a realistic percentile value. The thresholds are calculated separately for each time period and grid box.

### CTN90pct: Similar to CTX90pct, this definition employs the 90th percentile of T_min_ (minimum temperature) for each calendar day, following the same methodology as T_max_.

### EHF: The Excess Heat Factor, as defined by Nairn et al. (2009), is based on two excess heat indices (EHIs): [Excess Heat Days (EHD) and Excess Heat Sum (EHS)](Nairn et al., 2009):

The Excess Heat Factor (EHF) is calculated based on two components: EHI (accl.) and EHI (sig.), each describing different anomalies related to temperature. EHI (accl.) represents the acclimatization anomaly over a 3-day window compared to the preceding 30 days, while EHI (sig.) represents the anomaly against an extreme threshold for the same 3-day window. The formulation for EHI (accl.) is given by Equation (1):

$EHI(accl.)=\left[\left(T_{i}+T_{i-1}+T_{i-2}\right)/3\right]-\left[\left(T_{i-3}+...+T_{i-32}\right)/30\right]$ (S1)

Where T_i_ is the average daily temperature for day i, T30 is the average temperature over the preceding 30 days, and T95 is the 95th percentile temperature for the same time period. On the other hand, EHI (sig.) is derived from Equation (2):

$EHI(sig.)=\left[\left(T_{i}+T_{i-1}+T_{i-2}\right)/3\right]-T_{95}$ (S2)

Where T97.5 represents the 97.5th percentile temperature for the time period. Finally, the Excess Heat Factor (EHF) is obtained by combining Equations (S1) and (S2) and calculating the difference:

$EHF=\max\left[1,EHI(accl.)\right]\times EHI(sig.)$ (S3)

It is important to note that EHF for a particular day (day i) depends on the temperatures of the previous two days (days i-1 and i-2) since the average daily temperature is calculated within a 24-hour cycle (0900-0900 LT). Therefore, the temperature conditions in the 3-day window centered on that day influence the EHF for a specific day.

On the other hand, cold waves, which refer to periods of unusually cold temperatures, are determined in ClimPACT2 using the Excess Cold Factor (ECF) developed by Nairn and Fawcett in 2011 (Nairn & Fawcett, 2011). The ECF is similar in concept to the Excess Heat Factor (EHF) and is based on two excess cold indices (ECI) that capture different aspects related to cold temperatures: acclimatization to cold and climatological significance;

$ECI(accl.)=\left[\left(TM_{i}+TM_{i-1}+TM_{i-2}\right)/3\right]-\left[\left(TM_{i-3}+...+TM_{i-32}\right)/32\right]$ (S4)

$ECI(sig.)=\left[\left(TM_{i}+TM_{i-1}+TM_{i-2}\right)/3\right]-TM05$ (S5)

Where,

$ECF=-ECI(sig.)\times\min\left(-1,ECI(accl.)\right)$ (6S)

### In the ECF calculation, TM_i_ refers to the average daily temperature for day i, and TM05 represents the 5th percentile of the average daily temperature (TM) within a specified base period. The average daily temperature (TM) is obtained by taking the average of the maximum daily temperature (TX) and the minimum daily temperature (TN) (Nairn & Fawcett, 2011).

| **Table S2.** The results of the Mann-Kendall trend analysis (in variables of TX10p, TX90p, TN10p, and TN90p) across different identified clusters in Kazakhstan between 1959 and 2021. | | | | | | |
| --- | --- | --- | --- | --- | --- | --- |
| ***Cluster 1*** | | | | | | |
| **Variable** | **Location** | | **Maximum percentage** | **Minimum percentage** | **Sen's slope** | **P-value** |
| ***TX10p*** | Highest changes over time | Komsomolets | 23.1 (1976) | 2.1 (1988) | -0.13 | 0 |
|  | Lowest Changes over time | Sergeyevka | 24.1 (1969) | 1.8 (1988) | -0.07 | 0.017 |
| ***TX90p*** | Highest changes over time | Komsomolets | 26.8 (2020) | 2.2 (1959) | 0.21 | 0 |
|  | Lowest changes over time | Borovskoy | 23.8 (2020) | 2.7 (1959) | 0.09 | 0.007 |
| ***TN10p*** | Highest changes over time | Komsomolets | 23.3 (1969) | 2.1 (1988) | -0.14 | 0 |
|  | Lowest changes over time | Sergeyevka | 24.4 (1969) | 1.5 (1988) | -0.08 | 0.017 |
| ***TN90p*** | Highest changes over time | Komsomolets | 27.1 (2020) | 3.0 (1959) | 0.20 | 0 |
|  | Lowest changes over time | Borovskoy | 24.0 (2020) | 2.7 (1959) | 0.10 | 0.004 |
| ***Cluster 2*** | | | | | | |
| **Variable** | **Location** | | **Maximum percentage** | **Minimum percentage** | **Sen's slope** | **P-value** |
| ***TX10p*** | Highest changes over time | Aqqaytym | 24.3 (1976) | 1.6 (2021) | -0.16 | 0 |
|  | Lowest Changes over time | Shubarshi | 25.8 (1976) | 1.4 (2010) | -0.10 | 0 |
| ***TX90p*** | Highest changes over time | Temir | 29.0 (2010) | 3.0 (1959) | 0.27 | 0 |
|  | Lowest changes over time | Aral | 26.6 (2021) | 2.7 (1959) | 0.14 | 0 |
| ***TN10p*** | Highest changes over time | Aqqaytym | 24.4 (1976) | 1.9 (2021) | -0.16 | 0 |
|  | Lowest changes over time | Shubarshi | 25.9 (1976) | 1.6 (2010) | -0.11 | 0 |
| ***TN90p*** | Highest changes over time | Temir | 30.1 (2010) | 3.3 (1959) | 0.28 | 0 |
|  | Lowest changes over time | Aral | 26.3 (2021) | 2.7 (1959) | 0.20 | 0 |
| ***Cluster 3*** | | | | | | |
| **Variable** | **Location** | | **Maximum percentage** | **Minimum percentage** | **Sen's slope** | **P-value** |
| ***TX10p*** | Highest changes over time | Kokpekty | 27.9 (1960) | 3.6 (2020) | -0.15 | 0 |
|  | Lowest Changes over time | Ridder | 23.3 (1969) | 3.6 (2020) | -0.10 | 0 |
| ***TX90p*** | Highest changes over time | Shar | 23.3 (2002) | 2.5 (1960) | 0.23 | 0 |
|  | Lowest changes over time | Kurchum | 21.7 (1997) | 1.9 (1960) | 0.13 | 0 |
| ***TN10p*** | Highest changes over time | Kokpekty | 27.6 (1960) | 3.3 (2020) | -0.15 | 0 |
|  | Lowest changes over time | Ridder | 23.8 (1969) | 3.6 (2020) | -0.10 | 0 |
| ***TN90p*** | Highest changes over time | Shar | 22.7 (2002) | 3.3 (1960) | 0.23 | 0 |
|  | Lowest changes over time | Semey | 24.4 (2002) | 3.3 (1960) | 0.13 | 0 |
| ***Cluster 4*** | | | | | | |
| **Variable** | **Location** | | **Maximum percentage** | **Minimum percentage** | **Sen's slope** | **P-value** |
| ***TX10p*** | Highest changes over time | Saryozek | 18.6 (1960) | 3.3 (2019) | -0.15 | 0 |
|  | Lowest Changes over time | Merke | 22.7 (1972) | 3.7 (1990) | -0.07 | 0 |
| ***TX90p*** | Highest changes over time | Saryozek | 27.4 (2021) | 3.6 (1960) | 0.34 | 0 |
|  | Lowest changes over time | Zharkent | 29.6 (2021) | 4.1 (1964) | 0.08 | 0.029 |
| ***TN10p*** | Highest changes over time | Saryozek | 18.3 (1972) | 3.0 (2019) | -0.15 | 0 |
|  | Lowest changes over time | Merke | 22.8 (1972) | 3.2 (1990) | -0.07 | 0 |
| ***TN90p*** | Highest changes over time | Saryozek | 27.4 (2021) | 3.3 (1960) | 0.34 | 0 |
|  | Lowest changes over time | Zharkent | 29.9 (2021) | 4.6 (1964) | 0.07 | 0.059 |
| ***Cluster 5*** | | | | | | |
| **Variable** | **Location** | | **Maximum percentage** | **Minimum percentage** | **Sen's slope** | **P-value** |
| ***TX10p*** | Highest changes over time | Temirtau | 27.3 (1960) | 4.1 (2013) | -0.13 | 0 |
|  | Lowest Changes over time | Kurchatov | 25.2 (1969) | 3.3 (2020) | -0.07 | 0.026 |
| ***TX90p*** | Highest changes over time | Dzhambul | 25. 5 (2006) | 2.9 (1972) | 0.18 | 0 |
|  | Lowest changes over time | Kurchatov | 24.7 (2002) | 3.0 (1960) | 0.10 | 0.002 |
| ***TN10p*** | Highest changes over time | Temirtau | 27.3 (1960) | 4.1 (2013) | -0.13 | 0 |
|  | Lowest changes over time | Kurchatov | 25.2 (1969) | 3.6 (2020) | -0.07 | 0.023 |
| ***TN90p*** | Highest changes over time | Dzhambul | 24.7 (2006) | 2.7 (1972) | 0.18 | 0 |
|  | Lowest changes over time | Kurchatov | 23.6 (2002) | 3.0 (1960) | 0.10 | 0.003 |
| ***Cluster 6*** | | | | | | |
| **Variable** | **Location** | | **Maximum percentage** | **Minimum percentage** | **Sen's slope** | **P-value** |
| ***TX10p*** | Highest changes over time | Sarytoghay | 20.7 (1993) | 1.19 (2021) | -0.15 | 0 |
|  | Lowest Changes over time | Senek | 21.8 (1976) | 2.5 (2017) | -0.10 | 0 |
| ***TX90p*** | Highest changes over time | Zhanbay | 37.8 (2010) | 1.9 (1959) | 0.30 | 0 |
|  | Lowest changes over time | Fyodorovka | 24.6 (2021) | 2.7 (1959) | 0.12 | 0.001 |
| ***TN10p*** | Highest changes over time | Sarytoghay | 20.7 (1993) | 1.1 (2010) | -0.15 | 0 |
|  | Lowest changes over time | Senek | 22.1 (1976) | 2.5 (2017) | -0.10 | 0 |
| ***TN90p*** | Highest changes over time | Zhanbay | 37.3 (2010) | 1.9 (1959) | 0.30 | 0 |
|  | Lowest changes over time | Fyodorovka | 24.6 (2021) | 3.3 (1964) | 0.12 | 0.001 |
| ***Cluster 7*** | | | | | | |
| **Variable** | **Location** | | **Maximum percentage** | **Minimum percentage** | **Sen's slope** | **P-value** |
| ***TX10p*** | Highest changes over time | Taraz | 23.3 (1969) | 2.5 (2004) | -0.16 | 0 |
|  | Lowest Changes over time | Zhetysay | 24.7 (1969) | 4.1 (2015) | -0.06 | 0.006 |
| ***TX90p*** | Highest changes over time | Taraz | 34.0 (2021) | 2.2 (1960) | 0.35 | 0 |
|  | Lowest changes over time | Zhetysay | 24.4 (2021) | 6.1 (1992) | 0.12 | 0 |
| ***TN10p*** | Highest changes over time | Taraz | 23.6 (1969) | 2.2 (2019) | -0.20 | 0 |
|  | Lowest changes over time | Zhetysay | 23.8 (1969) | 4.1 (2015) | -0.06 | 0.01 |
| ***TN90p*** | Highest changes over time | Taraz | 33.7 (2021) | 2.7 (1960) | 0.36 | 0 |
|  | Lowest changes over time | Zhetysay | 25.2 (2021) | 6.2 (1992) | 0.11 | 0 |


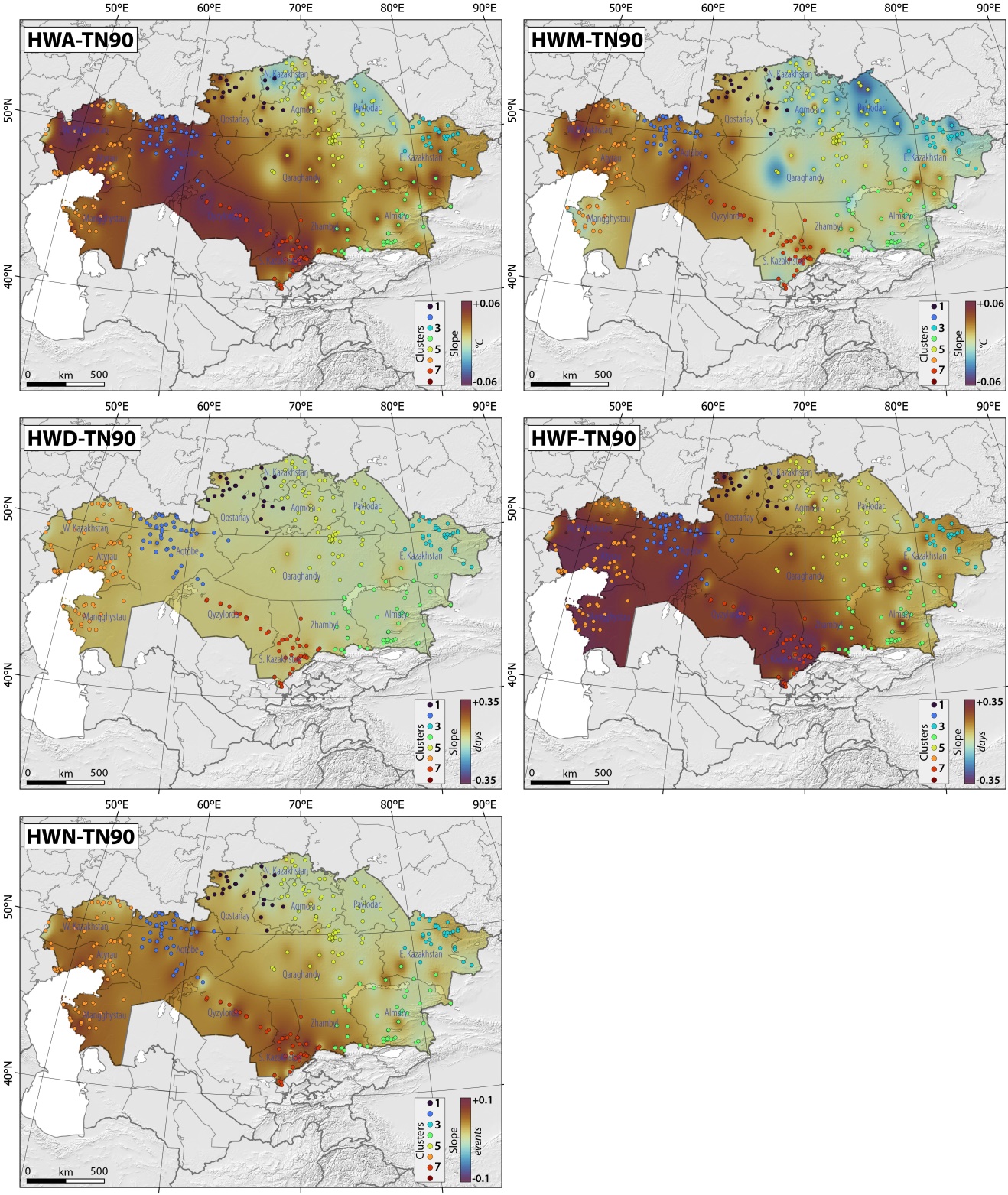


**Figure S1.** The slope of spatial-temporal changes in the HWA-TN90, HWM-TN90, HWD-TN90, HWF_-_TN90, and HWN-TN90 in Kazakhstan, Central Asia, between 1959 and 2021. Please note that colored dots and color scale refer to identified clusters and slope of changes, respectively, in Kazakhstan.


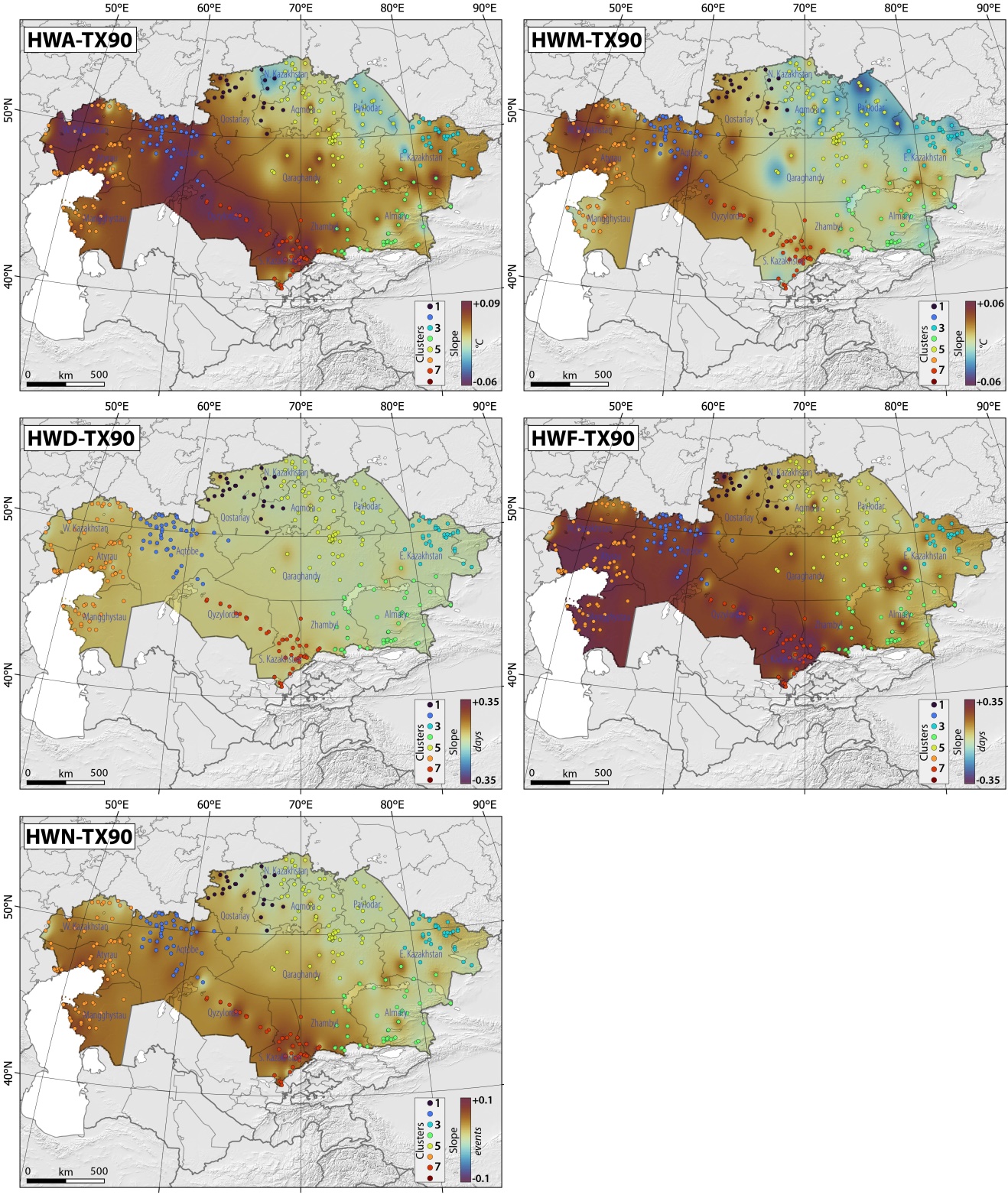


**Figure S2.** The slope of spatial-temporal changes in the HWA-TX90, HWM-TX90, HWD-TX90, HWF-TX90, and HWN-TX90 in Kazakhstan, Central Asia, between 1959 and 2021. Please note that colored dots and color scale refer to identified clusters and slope of changes, respectively, in Kazakhstan.

| ***Table S3.*** The results of the Mann-Kendall trend analysis (in variables of HWA, HWD, HWF, HWM, and HWN) across different identified clusters in Kazakhstan between 1959 and 2021. | | | | | |
| --- | --- | --- | --- | --- | --- |
| ***Cluster 1*** | | | | | |
| **Variable** | **Location** | | **Maximum Value** | **Sen's slope** | **P-value** |
| ***HWA*** | Highest Changes | Fyodorovka | 75.1 (2021) | 0.223 | 0.007 |
| ***HWD*** | Highest Changes | Komsomolets | 18 (1998) | 0.1 | 0.001 |
| ***HWF*** | Highest Changes | Komsomolets | 35 (2021) | 0.333 | 0 |
| ***HWM*** | Highest Changes | Fyodorovka | 31.2 (1963) | 0.072 | 0.022 |
| ***HWN*** | Highest Changes | Komsomolets | 6 (1995) | 0.045 | 0 |
| ***Cluster 2*** | | | | | |
| **Variable** | **Location** | | **Maximum Value** | **Sen's slope** | **P-value** |
| ***HWA*** | Highest Changes | Bel'kopa | 66.4 (2020) | 0.297 | 0.003 |
| ***HWD*** | Highest Changes | Aqkol | 15 (2016) | 0.136 | 0 |
| ***HWF*** | Highest Changes | Temir | 56 (2010) | 0.524 | 0 |
| ***HWM*** | Highest Changes | Aqkol | 31.1 (1990) | 0.069 | 0.005 |
| ***HWN*** | Highest Changes | Temir | 11 (2010) | 0.083 | 0 |
| ***Cluster 3*** | | | | | |
| **Variable** | **Location** | | **Maximum Value** | **Sen's slope** | **P-value** |
| ***HWA*** | Highest Changes | Ridder | 66 (2004) | 0.243 | 0.006 |
| ***HWD*** | Highest Changes | Shar | 11 (1966) | 0.08 | 0.001 |
| ***HWF*** | Highest Changes | Shar | 25 (1974) | 0.317 | 0 |
| ***HWM*** | Highest Changes | Ridder | 21 (2004) | 0.067 | 0.049 |
| ***HWN*** | Highest Changes | Shar | 6 (1974) | 0.045 | 0 |
| ***Cluster 4*** | | | | | |
| **Variable** | **Location** | | **Maximum Value** | **Sen's slope** | **P-value** |
| ***HWA*** | Highest Changes | Saryozek | 51.2 (1981) | 0.303 | 0.001 |
| ***HWD*** | Highest Changes | Saryozek | 13 (2015) | 0.119 | 0 |
| ***HWF*** | Highest Changes | Saryozek | 34 (2021) | 0.529 | 0 |
| ***HWM*** | Highest Changes | Sarykamys | 28.2 (1981) | 0.037 | 0.019 |
| ***HWN*** | Highest Changes | Saryozek | 6 (2021) | 0.096 | 0 |
| ***Cluster 5*** | | | | | |
| **Variable** | **Location** | | **Maximum Value** | **Sen's slope** | **P-value** |
| ***HWA*** | Highest Changes | Osakarovka | 98 (2004) | 0.345 | 0.002 |
| ***HWD*** | Highest Changes | Shubarkol | 14 (2003) | 0.1 | 0 |
| ***HWF*** | Highest Changes | Shubarkol | 26 (2012) | 0.25 | 0.001 |
| ***HWM*** | Highest Changes | Osakarovka | 35 (1988) | 0.082 | 0.059 |
| ***HWN*** | Highest Changes | Dzhambul | 6 (2008) | 0.036 | 0.001 |
| ***Cluster 6*** | | | | | |
| **Variable** | **Location** | | **Maximum Value** | **Sen's slope** | **P-value** |
| ***HWA*** | Highest Changes | Kaztalovka | 55.1 (2012) | 0.38 | 0.001 |
| ***HWD*** | Highest Changes | Mikil | 29 (2021) | 0.167 | 0 |
| ***HWF*** | Highest Changes | Mikil | 67 (2021) | 0.516 | 0 |
| ***HWM*** | Highest Changes | Tasqala | 15.7 (2019) | 0.112 | 0.002 |
| ***HWN*** | Highest Changes | Yeraliyev | 7 (2014, 2015, 2017, and 2018) | 0.067 | 0 |
| ***Cluster 7*** | | | | | |
| **Variable** | **Location** | | **Maximum Value** | **Sen's slope** | **P-value** |
| ***HWA*** | Highest Changes | Karatau | 49.3 (2020) | 0.399 | 0 |
| ***HWD*** | Highest Changes | Karatau | 18 (2019 and 2021) | 0.133 | 0 |
| ***HWF*** | Highest Changes | Taraz | 70 (2021) | 0.577 | 0 |
| ***HWM*** | Highest Changes | Temirlanovka | 12.1 (1970) | 0.065 | 0.136 |
| ***HWN*** | Highest Changes | Taraz | 10 (2000) | 0.093 | 0 |

| ***Table S4.*** The results of the Mann-Kendall trend analysis (in variables of CWA, CWD, CWF, CWM, and CWN) across different identified clusters in Kazakhstan between 1959 and 2021. | | | | | |
| --- | --- | --- | --- | --- | --- |
| ***Cluster 1*** | | | | | |
| **Variable** | **Location** | | **Maximum/ Minimum Value** | **Sen's slope** | **P-value** |
| ***CWA*** | Highest Changes | Tobyl | -388.0 (2006) | 1.316 | 0.042 |
| ***CWD*** | Highest Changes | Tobyl | 33.0 (1969) | -0.049 | 0.069 |
| ***CWF*** | Highest Changes | Fyodorovka | 41.0 (1969) | -0.118 | 0.027 |
| ***CWM*** | Highest Changes | Tobyl | -137.4 (2009) | 0.314 | 0.076 |
| ***CWN*** | Highest Changes | Komsomolets | 9.0 (2010) | -0.024 | 0.003 |
| ***Cluster 2*** | | | | | |
| **Variable** | **Location** | | **Maximum/ Minimum Value** | **Sen's slope** | **P-value** |
| ***CWA*** | Highest Changes | Shalkar | -243.0 (1972) | 1.02 | 0.014 |
| ***CWD*** | Highest Changes | Temir | 34.0 (1972) | -0.089 | 0.003 |
| ***CWF*** | Highest Changes | Bel'kopa | 44.0 (1969) | -0.263 | 0.001 |
| ***CWM*** | Highest Changes | Aqkol | -114.7 (2006) | 0.159 | 0.121 |
| ***CWN*** | Highest Changes | Shubarshi | 5.0 (1968) | -0.028 | 0.003 |
| ***Cluster 3*** | | | | | |
| **Variable** | **Location** | | **Maximum/ Minimum Value** | **Sen's slope** | **P-value** |
| ***CWA*** | Highest Changes | Boko | -578.0 (1966) | 0.933 | 0.24 |
| ***CWD*** | Highest Changes | Altayskiy | 29.0 (2012) | -0.115 | 0.001 |
| ***CWF*** | Highest Changes | Maleyevsk | 55.0 (1969) | -0.25 | 0.005 |
| ***CWM*** | Highest Changes | Serebryansk | -114.0 (1987) | 0.091 | 0.471 |
| ***CWN*** | Highest Changes | Shar | 6.0 (2010) | -0.025 | 0.004 |
| ***Cluster 4*** | | | | | |
| **Variable** | **Location** | | **Maximum/ Minimum Value** | **Sen's slope** | **P-value** |
| ***CWA*** | Highest Changes | Kapshagay | -459.0 (1984) | 1.45 | 0.026 |
| ***CWD*** | Highest Changes | Druzhba | 33.0 (1984) | -0.118 | 0.002 |
| ***CWF*** | Highest Changes | Ulken | 55.0 (1969) | -0.316 | 0 |
| ***CWM*** | Highest Changes | Qonayev | -120.2 (1959) | 0.274 | 0.07 |
| ***CWN*** | Highest Changes | Matay | 7.0 (1964) | -0.038 | 0 |
| ***Cluster 5*** | | | | | |
| **Variable** | **Location** | | **Maximum/ Minimum Value** | **Sen's slope** | **P-value** |
| ***CWA*** | Highest Changes | Shubarkol | -454.0 (2012) | 0.925 | 0.068 |
| ***CWD*** | Highest Changes | Temirtau | 34.0 (1969) | -0.067 | 0.005 |
| ***CWF*** | Highest Changes | Moyynty | 52.0 (1969) | -0.179 | 0.009 |
| ***CWM*** | Highest Changes | Ekibastuz | -184.1 (1987) | 0.075 | 0.74 |
| ***CWN*** | Highest Changes | Satbayev (city), Satpayev, and Zhezkazgan | 6.0 (1967) | -0.02 | 0.009 |
| ***Cluster 6*** | | | | | |
| **Variable** | **Location** | | **Maximum/ Minimum Value** | **Sen's slope** | **P-value** |
| ***CWA*** | Highest Changes | Kaztalovka | -317.1 (1972) | 1.364 | 0.009 |
| ***CWD*** | Highest Changes | Saykhin | 28.0 (1972) | -0.069 | 0.02 |
| ***CWF*** | Highest Changes | Saykhin | 52.0 (1969) | -0.227 | 0.002 |
| ***CWM*** | Highest Changes | Kaztalovka | -96.1 (2009) | 0.261 | 0.062 |
| ***CWN*** | Highest Changes | Suyindik | 6.0 (1997) | -0.037 | 0 |
| ***Cluster 7*** | | | | | |
| **Variable** | **Location** | | **Maximum/ Minimum Value** | **Sen's slope** | **P-value** |
| ***CWA*** | Highest Changes | Belkoel | -363.0 (1973) | 0.918 | 0.078 |
| ***CWD*** | Highest Changes | Asyqata | 36.0 (1969) | -0.095 | 0.021 |
| ***CWF*** | Highest Changes | Myrzakent | 68.0 (1972) | -0.3 | 0 |
| ***CWM*** | Highest Changes | Asyqata | -80.2 (1978) | 0.16 | 0.079 |
| ***CWN*** | Highest Changes | Zhanatas | 5.0 (1964, 1972, 1974, and 1984) | -0.036 | 0 |

| ***Table S5.*** The results of the Mann-Kendall trend analysis (CCD) across different identified clusters in Kazakhstan between 1959 and 2021. | | | | | |
| --- | --- | --- | --- | --- | --- |
| ***Cluster 1*** | | | | | |
| **Variable** | **Location** | | **Maximum Value** | **Sen's slope** | **P-value** |
| ***CCD*** | Highest increase | Komsomolets | 50.0 (2004) | 0.111 | 0.149 |
| ***Cluster 2*** | | | | | |
| **Variable** | **Location** | | **Maximum Value** | **Sen's slope** | **P-value** |
| ***CCD*** | Highest increase | Aqkol | 71.0 (1975) | 0.211 | 0.023 |
| ***Cluster 3*** | | | | | |
| **Variable** | **Location** | | **Maximum Value** | **Sen's slope** | **P-value** |
| ***CCD*** | Highest increase | Zaysan | 41.0 (2019) | 0.067 | 0.166 |
| ***Cluster 4*** | | | | | |
| **Variable** | **Location** | | **Maximum Value** | **Sen's slope** | **P-value** |
| ***CCD*** | Highest increase | Priozer | 100.0 (1980) | 0.053 | 0.618 |
| ***Cluster 5*** | | | | | |
| **Variable** | **Location** | | **Maximum Value** | **Sen's slope** | **P-value** |
| ***CCD*** | Highest increase | Shakhtinsk | 46.0 (1967) | 0.091 | 0.043 |
| ***Cluster 6*** | | | | | |
| **Variable** | **Location** | | **Maximum Value** | **Sen's slope** | **P-value** |
| ***CCD*** | Highest increase | Akshukyr | 98.0 (2018) | 0.385 | 0 |
| ***Cluster 7*** | | | | | |
| **Variable** | **Location** | | **Maximum Value** | **Sen's slope** | **P-value** |
| ***CCD*** | Highest increase | Zhetysay | 245.0 (1975) | 0.486 | 0.055 |

| **Table S6.** The correlation between HWN-EHF and CDD across different identified clusters in Kazakhstan between 1959 and 2021. | | |
| --- | --- | --- |
| ***Cluster 1*** | | |
| **Location** | **R^2^** | **P-value** |
| Lisakovsk | 0.14 | 0.27 |
| ***Cluster 2*** | | |
| **Location** | **R^2^** | **P-value** |
| Akrab | 0.46 | 0.00 |
| ***Cluster 3*** | | |
| **Location** | **R^2^** | **P-value** |
| Pervorossiyskoye | 0.28 | 0.03 |
| ***Cluster 4*** | | |
| **Location** | **R^2^** | **P-value** |
| Pervomayka | 0.40 | 0.00 |
| ***Cluster 5*** | | |
| **Location** | **R^2^** | **P-value** |
| Stepnyak | 0.27 | 0.03 |
| ***Cluster 6*** | | |
| **Location** | **R^2^** | **P-value** |
| Aqkol | 0.61 | 0.00 |
| ***Cluster 7*** | | |
| **Location** | **R^2^** | **P-value** |
| Zhosaly | 0.33 | 0.01 |

| **Table S7.** The number of all-cause mortalities associated with HWN-EHF in Kazakhstan from 1959 to 2021. | | | | | | | | | | | | | | | |
| --- | --- | --- | --- | --- | --- | --- | --- | --- | --- | --- | --- | --- | --- | --- | --- |
| **Year** | **Cluster 1** | **Cluster 2** | **Cluster 3** | **Cluster 4** | **Cluster 5** | **Cluster 6** | **Cluster 7** | **Year** | **Cluster 1** | **Cluster 2** | **Cluster 3** | **Cluster 4** | **Cluster 5** | **Cluster 6** | **Cluster 7** |
| **1959** | 0 | 0 | 0 | 4 | 5 | 2 | 9 | **1991** | 12 | 9 | 14 | 11 | 50 | 11 | 7 |
| **1960** | 2 | 2 | 0 | 0 | 3 | 5 | 4 | **1992** | 2 | 0 | 15 | 2 | 30 | 0 | 0 |
| **1961** | 9 | 3 | 0 | 12 | 19 | 11 | 28 | **1993** | 0 | 0 | 0 | 0 | 0 | 3 | 9 |
| **1962** | 9 | 11 | 13 | 13 | 29 | 9 | 20 | **1994** | 3 | 3 | 5 | 6 | 17 | 9 | 11 |
| **1963** | 8 | 2 | 2 | 1 | 21 | 8 | 6 | **1995** | 21 | 20 | 6 | 9 | 29 | 30 | 44 |
| **1964** | 0 | 2 | 2 | 0 | 2 | 9 | 1 | **1996** | 7 | 4 | 0 | 13 | 7 | 33 | 45 |
| **1965** | 7 | 2 | 14 | 21 | 60 | 3 | 37 | **1997** | 13 | 0 | 22 | 31 | 61 | 0 | 90 |
| **1966** | 6 | 5 | 9 | 10 | 41 | 16 | 38 | **1998** | 19 | 13 | 18 | 9 | 67 | 24 | 69 |
| **1967** | 1 | 12 | 2 | 2 | 12 | 14 | 4 | **1999** | 6 | 9 | 28 | 6 | 82 | 15 | 73 |
| **1968** | 10 | 9 | 10 | 2 | 25 | 12 | 6 | **2000** | 13 | 5 | 13 | 19 | 54 | 14 | 120 |
| **1969** | 3 | 0 | 11 | 2 | 26 | 12 | 6 | **2001** | 12 | 5 | 17 | 8 | 45 | 6 | 72 |
| **1970** | 3 | 1 | 0 | 2 | 22 | 5 | 7 | **2002** | 1 | 9 | 9 | 7 | 16 | 26 | 62 |
| **1971** | 11 | 12 | 2 | 0 | 9 | 19 | 16 | **2003** | 15 | 9 | 5 | 2 | 60 | 8 | 35 |
| **1972** | 0 | 2 | 0 | 1 | 0 | 27 | 1 | **2004** | 22 | 16 | 7 | 2 | 34 | 22 | 75 |
| **1973** | 0 | 2 | 0 | 3 | 8 | 0 | 7 | **2005** | 1 | 8 | 16 | 18 | 43 | 25 | 125 |
| **1974** | 5 | 2 | 25 | 4 | 13 | 8 | 8 | **2006** | 15 | 19 | 5 | 10 | 45 | 37 | 53 |
| **1975** | 8 | 17 | 6 | 12 | 13 | 23 | 32 | **2007** | 4 | 15 | 12 | 19 | 27 | 51 | 97 |
| **1976** | 4 | 9 | 5 | 1 | 21 | 10 | 18 | **2008** | 8 | 15 | 26 | 33 | 59 | 22 | 149 |
| **1977** | 8 | 4 | 8 | 12 | 43 | 13 | 25 | **2009** | 0 | 0 | 0 | 0 | 13 | 25 | 13 |
| **1978** | 6 | 5 | 6 | 7 | 32 | 1 | 8 | **2010** | 31 | 41 | 7 | 9 | 66 | 67 | 70 |
| **1979** | 2 | 6 | 0 | 1 | 6 | 10 | 16 | **2011** | 9 | 16 | 2 | 6 | 22 | 30 | 105 |
| **1980** | 8 | 2 | 5 | 0 | 28 | 1 | 18 | **2012** | 19 | 31 | 14 | 5 | 68 | 46 | 89 |
| **1981** | 8 | 2 | 11 | 2 | 32 | 11 | 6 | **2013** | 0 | 12 | 3 | 10 | 8 | 22 | 94 |
| **1982** | 6 | 3 | 13 | 20 | 35 | 16 | 38 | **2014** | 11 | 22 | 5 | 10 | 43 | 44 | 115 |
| **1983** | 8 | 6 | 0 | 9 | 20 | 13 | 43 | **2015** | 2 | 22 | 6 | 26 | 34 | 42 | 132 |
| **1984** | 2 | 7 | 0 | 20 | 18 | 18 | 42 | **2016** | 6 | 7 | 11 | 12 | 16 | 26 | 60 |
| **1985** | 0 | 5 | 3 | 13 | 10 | 6 | 6 | **2017** | 6 | 17 | 20 | 28 | 33 | 48 | 96 |
| **1986** | 0 | 1 | 7 | 16 | 8 | 11 | 38 | **2018** | 0 | 12 | 0 | 7 | 2 | 31 | 54 |
| **1987** | 5 | 8 | 8 | 8 | 21 | 12 | 12 | **2019** | 11 | 17 | 13 | 22 | 28 | 24 | 70 |
| **1988** | 8 | 13 | 4 | 6 | 33 | 17 | 16 | **2020** | 18 | 13 | 15 | 10 | 68 | 29 | 43 |
| **1989** | 14 | 8 | 2 | 0 | 41 | 13 | 16 | **2021** | 21 | 17 | 20 | 38 | 71 | 49 | 117 |
| **1990** | 3 | 5 | 5 | 10 | 11 | 8 | 16 |  |  |  |  |  |  |  |  |

| **Table S8.** The number of all-cause mortalities associated with HWN-TX90 in Kazakhstan from 1959 to 2021. | | | | | | | | | | | | | | | |
| --- | --- | --- | --- | --- | --- | --- | --- | --- | --- | --- | --- | --- | --- | --- | --- |
| **Year** | **Cluster 1** | **Cluster 2** | **Cluster 3** | **Cluster 4** | **Cluster 5** | **Cluster 6** | **Cluster 7** | **Year** | **Cluster 1** | **Cluster 2** | **Cluster 3** | **Cluster 4** | **Cluster 5** | **Cluster 6** | **Cluster 7** |
| **1959** | 0 | 0 | 3 | 4 | 4 | 8 | 10 | **1991** | 14 | 6 | 11 | 9 | 51 | 14 | 11 |
| **1960** | 2 | 4 | 0 | 0 | 3 | 5 | 8 | **1992** | 3 | 0 | 15 | 3 | 28 | 0 | 0 |
| **1961** | 8 | 7 | 0 | 10 | 18 | 15 | 36 | **1993** | 0 | 0 | 0 | 0 | 0 | 2 | 20 |
| **1962** | 9 | 10 | 15 | 12 | 36 | 13 | 21 | **1994** | 4 | 3 | 11 | 4 | 40 | 8 | 28 |
| **1963** | 6 | 2 | 5 | 1 | 24 | 8 | 8 | **1995** | 29 | 19 | 9 | 9 | 41 | 43 | 45 |
| **1964** | 0 | 4 | 2 | 0 | 0 | 8 | 10 | **1996** | 16 | 15 | 0 | 15 | 12 | 33 | 45 |
| **1965** | 9 | 4 | 11 | 21 | 68 | 5 | 37 | **1997** | 8 | 2 | 23 | 25 | 57 | 2 | 70 |
| **1966** | 8 | 5 | 9 | 10 | 45 | 23 | 44 | **1998** | 26 | 16 | 15 | 9 | 92 | 29 | 72 |
| **1967** | 2 | 16 | 2 | 1 | 17 | 17 | 16 | **1999** | 4 | 9 | 28 | 6 | 70 | 14 | 90 |
| **1968** | 12 | 6 | 11 | 2 | 21 | 12 | 7 | **2000** | 12 | 6 | 15 | 16 | 51 | 13 | 134 |
| **1969** | 4 | 0 | 12 | 5 | 42 | 9 | 4 | **2001** | 15 | 5 | 18 | 17 | 46 | 7 | 76 |
| **1970** | 4 | 3 | 0 | 2 | 16 | 3 | 9 | **2002** | 3 | 12 | 9 | 6 | 18 | 26 | 48 |
| **1971** | 8 | 12 | 0 | 1 | 6 | 22 | 19 | **2003** | 17 | 9 | 11 | 2 | 68 | 9 | 38 |
| **1972** | 2 | 2 | 0 | 0 | 10 | 26 | 4 | **2004** | 22 | 17 | 6 | 6 | 62 | 30 | 62 |
| **1973** | 2 | 0 | 0 | 3 | 10 | 1 | 9 | **2005** | 4 | 7 | 17 | 19 | 52 | 44 | 128 |
| **1974** | 8 | 5 | 19 | 9 | 20 | 8 | 23 | **2006** | 14 | 24 | 9 | 6 | 34 | 42 | 59 |
| **1975** | 9 | 18 | 6 | 11 | 18 | 26 | 25 | **2007** | 9 | 13 | 13 | 18 | 47 | 55 | 112 |
| **1976** | 7 | 10 | 2 | 4 | 29 | 10 | 17 | **2008** | 14 | 22 | 25 | 37 | 46 | 22 | 160 |
| **1977** | 8 | 5 | 11 | 15 | 50 | 16 | 27 | **2009** | 0 | 3 | 0 | 0 | 19 | 24 | 13 |
| **1978** | 9 | 5 | 6 | 6 | 40 | 0 | 10 | **2010** | 27 | 51 | 11 | 7 | 96 | 81 | 78 |
| **1979** | 4 | 7 | 0 | 0 | 0 | 17 | 17 | **2011** | 10 | 16 | 3 | 14 | 24 | 38 | 106 |
| **1980** | 6 | 5 | 6 | 6 | 31 | 2 | 20 | **2012** | 24 | 35 | 13 | 5 | 71 | 53 | 98 |
| **1981** | 10 | 2 | 11 | 4 | 34 | 10 | 1 | **2013** | 3 | 12 | 7 | 13 | 0 | 27 | 107 |
| **1982** | 7 | 6 | 18 | 21 | 55 | 15 | 33 | **2014** | 8 | 23 | 13 | 14 | 36 | 49 | 122 |
| **1983** | 10 | 7 | 3 | 7 | 15 | 10 | 41 | **2015** | 7 | 21 | 8 | 33 | 39 | 40 | 148 |
| **1984** | 4 | 10 | 0 | 17 | 28 | 22 | 45 | **2016** | 8 | 7 | 6 | 11 | 17 | 29 | 62 |
| **1985** | 2 | 5 | 3 | 9 | 9 | 12 | 12 | **2017** | 8 | 14 | 21 | 32 | 40 | 42 | 85 |
| **1986** | 0 | 3 | 5 | 11 | 9 | 5 | 23 | **2018** | 1 | 12 | 0 | 6 | 2 | 41 | 44 |
| **1987** | 8 | 10 | 13 | 8 | 16 | 20 | 23 | **2019** | 14 | 17 | 6 | 28 | 19 | 26 | 72 |
| **1988** | 11 | 14 | 8 | 4 | 30 | 18 | 14 | **2020** | 17 | 13 | 14 | 12 | 68 | 33 | 47 |
| **1989** | 13 | 8 | 5 | 2 | 41 | 14 | 16 | **2021** | 26 | 24 | 19 | 39 | 75 | 61 | 112 |
| **1990** | 4 | 4 | 8 | 16 | 13 | 5 | 23 |  |  |  |  |  |  |  |  |

| **Table S9.** The number of all-cause mortalities associated with HWN-TN90 in Kazakhstan from 1959 to 2021. | | | | | | | | | | | | | | | |
| --- | --- | --- | --- | --- | --- | --- | --- | --- | --- | --- | --- | --- | --- | --- | --- |
| **Year** | **Cluster 1** | **Cluster 2** | **Cluster 3** | **Cluster 4** | **Cluster 5** | **Cluster 6** | **Cluster 7** | **Year** | **Cluster 1** | **Cluster 2** | **Cluster 3** | **Cluster 4** | **Cluster 5** | **Cluster 6** | **Cluster 7** |
| **1959** | 0 | 0 | 1 | 0 | 2 | 1 | 3 | **1991** | 2 | 1 | 1 | 1 | 12 | 2 | 2 |
| **1960** | 0 | 0 | 0 | 0 | 1 | 0 | 1 | **1992** | 1 | 0 | 2 | 0 | 6 | 0 | 0 |
| **1961** | 1 | 1 | 0 | 1 | 3 | 3 | 7 | **1993** | 0 | 0 | 0 | 0 | 0 | 0 | 3 |
| **1962** | 2 | 2 | 2 | 2 | 7 | 2 | 4 | **1994** | 1 | 1 | 2 | 0 | 8 | 1 | 5 |
| **1963** | 1 | 1 | 1 | 0 | 6 | 1 | 1 | **1995** | 5 | 4 | 1 | 1 | 8 | 8 | 8 |
| **1964** | 0 | 1 | 0 | 0 | 0 | 0 | 3 | **1996** | 2 | 1 | 0 | 1 | 3 | 6 | 8 |
| **1965** | 1 | 1 | 2 | 1 | 13 | 1 | 6 | **1997** | 1 | 0 | 5 | 2 | 11 | 1 | 12 |
| **1966** | 1 | 1 | 1 | 2 | 10 | 5 | 7 | **1998** | 5 | 4 | 4 | 1 | 19 | 6 | 13 |
| **1967** | 0 | 2 | 1 | 0 | 3 | 3 | 3 | **1999** | 0 | 1 | 5 | 0 | 14 | 4 | 18 |
| **1968** | 1 | 1 | 2 | 0 | 3 | 4 | 2 | **2000** | 1 | 1 | 2 | 2 | 12 | 4 | 27 |
| **1969** | 0 | 0 | 2 | 0 | 9 | 1 | 1 | **2001** | 2 | 1 | 3 | 3 | 9 | 2 | 16 |
| **1970** | 1 | 1 | 0 | 0 | 3 | 0 | 2 | **2002** | 0 | 2 | 2 | 0 | 4 | 6 | 9 |
| **1971** | 1 | 2 | 0 | 0 | 1 | 4 | 3 | **2003** | 2 | 1 | 2 | 0 | 15 | 2 | 7 |
| **1972** | 0 | 0 | 1 | 0 | 2 | 6 | 0 | **2004** | 4 | 3 | 1 | 0 | 14 | 6 | 11 |
| **1973** | 0 | 0 | 0 | 0 | 2 | 0 | 2 | **2005** | 1 | 1 | 4 | 3 | 10 | 8 | 30 |
| **1974** | 1 | 2 | 5 | 0 | 2 | 2 | 4 | **2006** | 3 | 5 | 2 | 0 | 8 | 8 | 9 |
| **1975** | 1 | 3 | 1 | 0 | 2 | 4 | 4 | **2007** | 1 | 3 | 3 | 0 | 10 | 11 | 22 |
| **1976** | 1 | 2 | 1 | 0 | 5 | 2 | 3 | **2008** | 1 | 4 | 4 | 4 | 10 | 4 | 33 |
| **1977** | 1 | 1 | 1 | 1 | 11 | 4 | 4 | **2009** | 0 | 1 | 0 | 0 | 4 | 4 | 3 |
| **1978** | 1 | 1 | 2 | 0 | 9 | 0 | 1 | **2010** | 7 | 12 | 2 | 2 | 18 | 14 | 13 |
| **1979** | 1 | 1 | 0 | 0 | 0 | 2 | 3 | **2011** | 1 | 3 | 0 | 0 | 6 | 8 | 19 |
| **1980** | 1 | 1 | 1 | 0 | 7 | 0 | 4 | **2012** | 5 | 7 | 3 | 0 | 15 | 11 | 17 |
| **1981** | 1 | 1 | 2 | 1 | 7 | 2 | 1 | **2013** | 1 | 2 | 1 | 0 | 0 | 5 | 20 |
| **1982** | 1 | 1 | 3 | 2 | 12 | 4 | 6 | **2014** | 2 | 5 | 1 | 2 | 8 | 10 | 23 |
| **1983** | 1 | 1 | 1 | 0 | 4 | 1 | 8 | **2015** | 2 | 4 | 2 | 5 | 8 | 8 | 31 |
| **1984** | 0 | 2 | 0 | 1 | 5 | 5 | 8 | **2016** | 1 | 1 | 1 | 2 | 4 | 6 | 12 |
| **1985** | 0 | 1 | 1 | 1 | 2 | 1 | 2 | **2017** | 1 | 2 | 5 | 4 | 8 | 8 | 18 |
| **1986** | 0 | 1 | 1 | 1 | 3 | 0 | 4 | **2018** | 0 | 3 | 0 | 1 | 0 | 8 | 7 |
| **1987** | 1 | 2 | 3 | 2 | 5 | 4 | 5 | **2019** | 2 | 3 | 1 | 2 | 6 | 6 | 15 |
| **1988** | 1 | 2 | 1 | 0 | 5 | 3 | 2 | **2020** | 2 | 2 | 2 | 2 | 15 | 5 | 10 |
| **1989** | 2 | 1 | 1 | 0 | 8 | 3 | 2 | **2021** | 5 | 4 | 4 | 5 | 15 | 13 | 22 |
| **1990** | 1 | 1 | 1 | 0 | 3 | 0 | 4 |  |  |  |  |  |  |  |  |

| **Table S10.** The number of CVD mortality associated with HWN-EHF in Kazakhstan from 1959 to 2021. | | | | | | | | | | | | | | | |
| --- | --- | --- | --- | --- | --- | --- | --- | --- | --- | --- | --- | --- | --- | --- | --- |
| **Year** | **Cluster 1** | **Cluster 2** | **Cluster 3** | **Cluster 4** | **Cluster 5** | **Cluster 6** | **Cluster 7** | **Year** | **Cluster 1** | **Cluster 2** | **Cluster 3** | **Cluster 4** | **Cluster 5** | **Cluster 6** | **Cluster 7** |
| **1990** | 1 | 1 | 1 | 0 | 2 | 2 | 3 | **2005** | 0 | 2 | 6 | 3 | 17 | 7 | 40 |
| **1991** | 3 | 2 | 3 | 1 | 12 | 3 | 2 | **2006** | 6 | 6 | 1 | 2 | 16 | 13 | 15 |
| **1992** | 1 | 0 | 4 | 0 | 7 | 0 | 0 | **2007** | 1 | 4 | 4 | 5 | 10 | 15 | 30 |
| **1993** | 0 | 0 | 0 | 0 | 0 | 1 | 2 | **2008** | 3 | 5 | 7 | 6 | 18 | 7 | 44 |
| **1994** | 1 | 1 | 1 | 2 | 4 | 3 | 3 | **2009** | 0 | 0 | 0 | 0 | 4 | 8 | 4 |
| **1995** | 5 | 5 | 1 | 2 | 8 | 8 | 12 | **2010** | 9 | 13 | 3 | 1 | 19 | 17 | 20 |
| **1996** | 3 | 1 | 0 | 4 | 2 | 10 | 11 | **2011** | 2 | 4 | 0 | 0 | 5 | 9 | 32 |
| **1997** | 4 | 0 | 7 | 5 | 20 | 0 | 25 | **2012** | 6 | 10 | 5 | 0 | 22 | 13 | 26 |
| **1998** | 5 | 3 | 5 | 1 | 19 | 6 | 18 | **2013** | 0 | 3 | 1 | 1 | 2 | 6 | 26 |
| **1999** | 1 | 2 | 8 | 0 | 22 | 4 | 19 | **2014** | 2 | 6 | 1 | 1 | 11 | 12 | 31 |
| **2000** | 3 | 1 | 4 | 3 | 16 | 5 | 35 | **2015** | 0 | 6 | 2 | 5 | 9 | 11 | 39 |
| **2001** | 2 | 1 | 5 | 1 | 15 | 3 | 21 | **2016** | 1 | 2 | 3 | 2 | 5 | 8 | 15 |
| **2002** | 0 | 2 | 2 | 1 | 3 | 9 | 19 | **2017** | 1 | 5 | 6 | 5 | 9 | 12 | 29 |
| **2003** | 6 | 2 | 1 | 0 | 22 | 2 | 9 | **2018** | 0 | 3 | 0 | 1 | 1 | 8 | 16 |
| **2004** | 7 | 4 | 1 | 0 | 13 | 6 | 25 | **2019** | 3 | 5 | 4 | 4 | 8 | 7 | 23 |

| **Table S11.** The number of CVD mortality associated with HWN-TX90 in Kazakhstan from 1959 to 2021. | | | | | | | | | | | | | | | |
| --- | --- | --- | --- | --- | --- | --- | --- | --- | --- | --- | --- | --- | --- | --- | --- |
| **Year** | **Cluster 1** | **Cluster 2** | **Cluster 3** | **Cluster 4** | **Cluster 5** | **Cluster 6** | **Cluster 7** | **Year** | **Cluster 1** | **Cluster 2** | **Cluster 3** | **Cluster 4** | **Cluster 5** | **Cluster 6** | **Cluster 7** |
| **1990** | 1 | 1 | 2 | 1 | 3 | 2 | 5 | **2005** | 1 | 2 | 6 | 3 | 19 | 14 | 41 |
| **1991** | 4 | 1 | 4 | 1 | 14 | 4 | 3 | **2006** | 6 | 7 | 2 | 1 | 12 | 13 | 17 |
| **1992** | 1 | 0 | 4 | 0 | 6 | 0 | 0 | **2007** | 3 | 4 | 4 | 3 | 17 | 16 | 35 |
| **1993** | 0 | 0 | 0 | 0 | 0 | 1 | 5 | **2008** | 5 | 7 | 5 | 8 | 13 | 6 | 51 |
| **1994** | 1 | 1 | 2 | 0 | 12 | 3 | 6 | **2009** | 0 | 1 | 0 | 0 | 6 | 8 | 4 |
| **1995** | 9 | 5 | 2 | 2 | 14 | 12 | 13 | **2010** | 8 | 17 | 4 | 1 | 30 | 21 | 22 |
| **1996** | 5 | 4 | 0 | 3 | 4 | 9 | 11 | **2011** | 3 | 4 | 1 | 2 | 6 | 10 | 32 |
| **1997** | 2 | 0 | 7 | 4 | 18 | 1 | 19 | **2012** | 7 | 11 | 4 | 0 | 22 | 14 | 30 |
| **1998** | 7 | 4 | 4 | 1 | 27 | 7 | 20 | **2013** | 1 | 3 | 2 | 2 | 0 | 8 | 30 |
| **1999** | 0 | 2 | 8 | 1 | 22 | 4 | 26 | **2014** | 1 | 7 | 3 | 2 | 10 | 12 | 36 |
| **2000** | 3 | 2 | 3 | 3 | 14 | 4 | 40 | **2015** | 2 | 5 | 3 | 7 | 10 | 12 | 44 |
| **2001** | 3 | 1 | 6 | 3 | 14 | 3 | 22 | **2016** | 2 | 2 | 1 | 2 | 5 | 7 | 16 |
| **2002** | 0 | 3 | 2 | 1 | 4 | 7 | 14 | **2017** | 1 | 4 | 6 | 7 | 11 | 11 | 25 |
| **2003** | 6 | 2 | 2 | 0 | 24 | 3 | 10 | **2018** | 0 | 3 | 0 | 1 | 1 | 10 | 11 |
| **2004** | 7 | 5 | 1 | 1 | 22 | 9 | 18 | **2019** | 5 | 5 | 2 | 6 | 5 | 7 | 22 |

| **Table S12.** The number of CVD mortality associated with HWN-TN90 in Kazakhstan from 1959 to 2021. | | | | | | | | | | | | | | | |
| --- | --- | --- | --- | --- | --- | --- | --- | --- | --- | --- | --- | --- | --- | --- | --- |
| **Year** | **Cluster 1** | **Cluster 2** | **Cluster 3** | **Cluster 4** | **Cluster 5** | **Cluster 6** | **Cluster 7** | **Year** | **Cluster 1** | **Cluster 2** | **Cluster 3** | **Cluster 4** | **Cluster 5** | **Cluster 6** | **Cluster 7** |
| **1990** | 1 | 1 | 2 | 1 | 3 | 2 | 5 | **2005** | 1 | 2 | 6 | 3 | 18 | 14 | 52 |
| **1991** | 4 | 1 | 3 | 1 | 15 | 4 | 2 | **2006** | 6 | 7 | 2 | 1 | 14 | 13 | 18 |
| **1992** | 1 | 0 | 4 | 0 | 6 | 0 | 0 | **2007** | 3 | 5 | 4 | 3 | 18 | 16 | 35 |
| **1993** | 0 | 0 | 0 | 0 | 0 | 1 | 5 | **2008** | 4 | 7 | 5 | 9 | 13 | 6 | 51 |
| **1994** | 1 | 1 | 2 | 0 | 12 | 3 | 6 | **2009** | 0 | 1 | 0 | 0 | 4 | 7 | 4 |
| **1995** | 8 | 5 | 2 | 2 | 14 | 11 | 13 | **2010** | 8 | 17 | 5 | 2 | 28 | 22 | 22 |
| **1996** | 4 | 2 | 0 | 3 | 4 | 9 | 11 | **2011** | 3 | 4 | 1 | 2 | 6 | 11 | 32 |
| **1997** | 3 | 0 | 7 | 4 | 18 | 1 | 19 | **2012** | 7 | 11 | 4 | 0 | 21 | 14 | 30 |
| **1998** | 6 | 5 | 4 | 1 | 27 | 7 | 20 | **2013** | 1 | 3 | 2 | 1 | 0 | 8 | 31 |
| **1999** | 1 | 2 | 7 | 0 | 21 | 4 | 26 | **2014** | 2 | 7 | 2 | 2 | 10 | 12 | 36 |
| **2000** | 3 | 1 | 3 | 3 | 15 | 4 | 39 | **2015** | 3 | 5 | 2 | 7 | 10 | 12 | 41 |
| **2001** | 3 | 2 | 6 | 3 | 15 | 3 | 23 | **2016** | 3 | 2 | 2 | 2 | 5 | 8 | 17 |
| **2002** | 0 | 3 | 2 | 1 | 4 | 8 | 13 | **2017** | 1 | 4 | 6 | 8 | 10 | 12 | 26 |
| **2003** | 6 | 2 | 2 | 0 | 24 | 3 | 11 | **2018** | 0 | 4 | 0 | 1 | 1 | 10 | 11 |
| **2004** | 7 | 5 | 1 | 0 | 22 | 9 | 20 | **2019** | 5 | 5 | 3 | 5 | 6 | 7 | 22 |

| **Table S13.** The Mann-Kendall trend analysis for all-cause and CVD mortalities associated with different definitions of heatwaves in Kazakhstan from 1959 to 2021. | | | | | | | |
| --- | --- | --- | --- | --- | --- | --- | --- |
| **All-cause mortality _HWN-EHF_** | ***Cluster1*** | ***Cluster 2*** | ***Cluster 3*** | ***Cluster 4*** | ***Cluster 5*** | ***Cluster 6*** | ***Cluster 7*** |
| p-value (Two-tailed) | **0.03** | **< 0.0001** | **0.01** | **0.00** | **0.00** | **< 0.0001** | **< 0.0001** |
| Sen’s slope | 0.09 | 0.21 | 0.12 | 0.17 | 0.46 | 0.45 | 1.44 |
| **All-cause mortality_HWN-TN90_** | ***Cluster1*** | ***Cluster 2*** | ***Cluster 3*** | ***Cluster 4*** | ***Cluster 5*** | ***Cluster 6*** | ***Cluster 7*** |
| p-value (Two-tailed) | **0.00** | **< 0.0001** | **0.01** | **0.00** | **0.00** | **< 0.0001** | **< 0.0001** |
| Sen’s slope | 0.00 | 0.03 | 0.00 | 0.00 | 0.11 | 0.11 | 0.25 |
| **All-cause mortality_HWN-TX90_** | ***Cluster1*** | ***Cluster 2*** | ***Cluster 3*** | ***Cluster 4*** | ***Cluster 5*** | ***Cluster 6*** | ***Cluster 7*** |
| p-value (Two-tailed) | **0.00** | **< 0.0001** | **0.01** | **0.00** | **0.01** | **< 0.0001** | **< 0.0001** |
| Sen’s slope | 0.13 | 0.21 | 0.14 | 0.19 | 0.46 | 0.50 | 1.34 |
| **CVD mortality _HWN-EHF_** | ***Cluster1*** | ***Cluster 2*** | ***Cluster 3*** | ***Cluster 4*** | ***Cluster 5*** | ***Cluster 6*** | ***Cluster 7*** |
| p-value (Two-tailed) | 0.62 | **0.00** | 0.96 | 0.19 | 0.94 | **0.00** | **0.00** |
| Sen’s slope | 0.00 | 0.17 | 0.00 | 0.00 | 0.00 | 0.32 | 0.88 |
| **CVD mortality_HWN-TN90_** | ***Cluster1*** | ***Cluster 2*** | ***Cluster 3*** | ***Cluster 4*** | ***Cluster 5*** | ***Cluster 6*** | ***Cluster 7*** |
| p-value (Two-tailed) | 0.94 | **0.00** | 0.77 | 0.08 | 0.52 | **0.00** | **0.00** |
| Sen’s slope | 0.00 | 0.15 | 0.00 | 0.05 | -0.13 | 0.32 | 0.94 |
| **CVD mortality_HWN-TX90_** | ***Cluster1*** | ***Cluster 2*** | ***Cluster 3*** | ***Cluster 4*** | ***Cluster 5*** | ***Cluster 6*** | ***Cluster 7*** |
| p-value (Two-tailed) | 0.93 | **0.00** | 0.56 | 0.06 | 0.46 | **0.00** | **0.00** |
| Sen’s slope | 0.00 | 0.14 | 0.00 | 0.06 | -0.13 | 0.31 | 0.92 |

| **Table S14.** The results of the Mann-Kendall trend analysis (CDD18 and HDD10) across different identified clusters in Kazakhstan between 1959 and 2021. | | | | |
| --- | --- | --- | --- | --- |
| ***Cluster 1*** | | | | |
| **Variable** | **Location** | | **Sen's slope** | **P-value** |
| ***CDD18*** | Highest Changes | Komsomolets | 4.145 | 0 |
|  | Lowest Changes | Sergeyevka | 0.671 | 0.209 |
| ***HDD10*** | Highest Changes | Derzhavinsk | -9.281 | 0 |
|  | Lowest Changes | Troyebratskiy | -7.356 | 0.002 |
| ***Cluster 2*** | | | | |
| **Variable** | **Location** | | **Sen's slope** | **P-value** |
| ***CDD18*** | Highest Changes | Temir | 5.238 | 0 |
|  | Lowest Changes | Aral | 2.545 | 0 |
| ***HDD10*** | Highest Changes | Aqqum | -10.521 | 0 |
|  | Lowest Changes | Aral | -8.352 | 0 |
| ***Cluster 3*** | | | | |
| **Variable** | **Location** | | **Sen's slope** | **P-value** |
| ***CDD18*** | Highest Changes | Shar | 4.084 | 0 |
|  | Lowest Changes | Ridder | 0.142 | 0.022 |
| ***HDD10*** | Highest Changes | Oskemen | -12.462 | 0 |
|  | Lowest Changes | Semey | -8.833 | 0.001 |
| ***Cluster 4*** | | | | |
| **Variable** | **Location** | | **Sen's slope** | **P-value** |
| ***CDD18*** | Highest Changes | Saryozek | 5.602 | 0 |
|  | Lowest Changes | Zharkent | 0.354 | 0.537 |
| ***HDD10*** | Highest Changes | Sarykamys | -10.295 | 0 |
|  | Lowest Changes | Zharkent | -5.167 | 0 |
| ***Cluster 5*** | | | | |
| **Variable** | **Location** | | **Sen's slope** | **P-value** |
| ***CDD18*** | Highest Changes | Shubarkol | 3.037 | 0 |
|  | Lowest Changes | Prigorodnoye | 0.151 | 0.678 |
| ***HDD10*** | Highest Changes | Zhezkazgan | -9.911 | 0 |
|  | Lowest Changes | Kurchatov | -6.25 | 0.009 |
| ***Cluster 6*** | | | | |
| **Variable** | **Location** | | **Sen's slope** | **P-value** |
| ***CDD18*** | Highest Changes | Yeraliyev | 5.362 | 0 |
|  | Lowest Changes | Fyodorovka | 1.357 | 0.03 |
| ***HDD10*** | Highest Changes | Aqkol | -10.442 | 0 |
|  | Lowest Changes | Bautino | -3.047 | 0.005 |
| ***Cluster 7*** | | | | |
| **Variable** | **Location** | | **Sen's slope** | **P-value** |
| ***CDD18*** | Highest Changes | Taraz | 6.043 | 0 |
|  | Lowest Changes | Temirlanovka | 0.959 | 0.037 |
| ***HDD10*** | Highest Changes | Taraz | -10.949 | 0 |
|  | Lowest Changes | Zhetysay | -3.781 | 0.01 |

| **Table S15.** The energy demand (kWh) analysis and associated CO_2_ emissions (kg/kWh) for heating and cooling purposes in different clusters in Kazakhstan between 1959 and 2021. | | | | | | | | | |
| --- | --- | --- | --- | --- | --- | --- | --- | --- | --- |
| ***Coal fired plant*** | | | | | | | | | |
| **Cluster** | **HDD10** (degree days) | **CDD18**  (degree days) | **Energy generation**  **for heating** | **Energy generation for cooling** | **Total Energy generation** | | **CO_2_ emission for heating** | **CO_2_ emission for cooling** | **Total CO_2_ emission** |
| 1 | 3450 | 271 | 82.81 | 6.51 | 89.32 | | 79.50 | 6.25 | 85.74 |
| 2 | 3018 | 497 | 72.43 | 11.92 | 84.35 | | 69.54 | 11.44 | 80.98 |
| 3 | 3528 | 229 | 84.67 | 5.50 | 90.17 | | 81.28 | 5.28 | 86.57 |
| 4 | 2421 | 495 | 58.09 | 11.87 | 69.97 | | 55.77 | 11.40 | 67.17 |
| 5 | 3460 | 264 | 83.04 | 6.34 | 89.38 | | 79.72 | 6.08 | 85.81 |
| 6 | 2035 | 807 | 48.85 | 19.36 | 68.21 | | 46.89 | 18.59 | 65.48 |
| 7 | 1641 | 889 | 39.38 | 21.32 | 60.70 | | 37.80 | 20.47 | 58.27 |
| ***Gas fired plant*** | | | | | | | | | |
| **Cluster** | **HDD10** (degree days) | **CDD18**  (degree days) | **Energy generation**  **for heating** | **Energy generation for cooling** | **Total Energy generation** | **CO_2_ emission for heating** | | **CO_2_ emission for cooling** | **Total CO_2_ emission** |
| 1 | 3450 | 271 | 82.81 | 6.51 | 89.32 | 71.96 | | 5.66 | 77.62 |
| 2 | 3018 | 497 | 72.43 | 11.92 | 84.35 | 62.95 | | 10.36 | 73.30 |
| 3 | 3528 | 229 | 84.67 | 5.50 | 90.17 | 73.58 | | 4.78 | 78.36 |
| 4 | 2421 | 495 | 58.09 | 11.87 | 69.97 | 50.48 | | 10.32 | 60.80 |
| 5 | 3460 | 264 | 83.04 | 6.34 | 89.38 | 72.16 | | 5.51 | 77.67 |
| 6 | 2035 | 807 | 48.85 | 19.36 | 68.21 | 42.45 | | 16.83 | 59.27 |
| 7 | 1641 | 889 | 39.38 | 21.32 | 60.70 | 34.22 | | 18.53 | 52.75 |
| ***Oil fired plant*** | | | | | | | | | |
| **Cluster** | **HDD10** (degree days) | **CDD18**  (degree days) | **Energy generation**  **for heating** | **Energy generation for cooling** | **Total Energy generation** | **CO_2_ emission for heating** | | **CO_2_ emission for cooling** | **Total CO_2_ emission** |
| 1 | 3450 | 271 | 82.81 | 6.51 | 89.32 | 49.35 | | 3.88 | 53.23 |
| 2 | 3018 | 497 | 72.43 | 11.92 | 84.35 | 43.17 | | 7.10 | 50.27 |
| 3 | 3528 | 229 | 84.67 | 5.50 | 90.17 | 50.46 | | 3.28 | 53.74 |
| 4 | 2421 | 495 | 58.09 | 11.87 | 69.97 | 34.62 | | 7.08 | 41.70 |
| 5 | 3460 | 264 | 83.04 | 6.34 | 89.38 | 49.49 | | 3.78 | 53.27 |
| 6 | 2035 | 807 | 48.85 | 19.36 | 68.21 | 29.11 | | 11.54 | 40.65 |
| 7 | 1641 | 889 | 39.38 | 21.32 | 60.70 | 23.47 | | 12.71 | 36.18 |
| ***Combined cycle gas*** | | | | | | | | | |
| **Cluster** | **HDD10** (degree days) | **CDD18**  (degree days) | **Energy generation**  **for heating** | **Energy generation for cooling** | **Total Energy generation** | **CO_2_ emission for heating** | | **CO_2_ emission for cooling** | **Total CO_2_ emission** |
| 1 | 3450 | 271 | 82.81 | 6.51 | 89.32 | 37.26 | | 2.93 | 40.19 |
| 2 | 3018 | 497 | 72.43 | 11.92 | 84.35 | 32.60 | | 5.36 | 37.96 |
| 3 | 3528 | 229 | 84.67 | 5.50 | 90.17 | 38.10 | | 2.48 | 40.58 |
| 4 | 2421 | 495 | 58.09 | 11.87 | 69.97 | 26.14 | | 5.34 | 31.48 |
| 5 | 3460 | 264 | 83.04 | 6.34 | 89.38 | 37.37 | | 2.85 | 40.22 |
| 6 | 2035 | 807 | 48.85 | 19.36 | 68.21 | 21.98 | | 8.71 | 30.69 |
| 7 | 1641 | 889 | 39.38 | 21.32 | 60.70 | 17.72 | | 9.60 | 27.32 |
| ***Hydroelectric*** | | | | | | | | | |
| **Cluster** | **HDD10** (degree days) | **CDD18**  (degree days) | **Energy generation**  **for heating** | **Energy generation for cooling** | **Total Energy generation** | **CO_2_ emission for heating** | | **CO_2_ emission for cooling** | **Total CO_2_ emission** |
| 1 | 3450 | 271 | 82.81 | 6.51 | 89.32 | 0.33 | | 0.03 | 0.36 |
| 2 | 3018 | 497 | 72.43 | 11.92 | 84.35 | 0.29 | | 0.05 | 0.34 |
| 3 | 3528 | 229 | 84.67 | 5.50 | 90.17 | 0.34 | | 0.02 | 0.36 |
| 4 | 2421 | 495 | 58.09 | 11.87 | 69.97 | 0.23 | | 0.05 | 0.28 |
| 5 | 3460 | 264 | 83.04 | 6.34 | 89.38 | 0.33 | | 0.03 | 0.36 |
| 6 | 2035 | 807 | 48.85 | 19.36 | 68.21 | 0.20 | | 0.08 | 0.27 |
| 7 | 1641 | 889 | 39.38 | 21.32 | 60.70 | 0.16 | | 0.09 | 0.24 |
| ***PV*** | | | | | | | | | |
| **Cluster** | **HDD10** (degree days) | **CDD18**  (degree days) | **Energy generation**  **for heating** | **Energy generation for cooling** | **Total Energy generation** | **CO_2_ emission for heating** | | **CO_2_ emission for cooling** | **Total CO_2_ emission** |
| 1 | 3450 | 271 | 82.81 | 6.51 | 89.32 | 8.28 | | 0.65 | 8.93 |
| 2 | 3018 | 497 | 72.43 | 11.92 | 84.35 | 7.24 | | 1.19 | 8.44 |
| 3 | 3528 | 229 | 84.67 | 5.50 | 90.17 | 8.47 | | 0.55 | 9.02 |
| 4 | 2421 | 495 | 58.09 | 11.87 | 69.97 | 5.81 | | 1.19 | 7.00 |
| 5 | 3460 | 264 | 83.04 | 6.34 | 89.38 | 8.30 | | 0.63 | 8.94 |
| 6 | 2035 | 807 | 48.85 | 19.36 | 68.21 | 4.88 | | 1.94 | 6.82 |
| 7 | 1641 | 889 | 39.38 | 21.32 | 60.70 | 3.94 | | 2.13 | 6.07 |
| ***Wind*** | | | | | | | | | |
| **Cluster** | **HDD10** (degree days) | **CDD18**  (degree days) | **Energy generation**  **for heating** | **Energy generation for cooling** | **Total Energy generation** | **CO_2_ emission for heating** | | **CO_2_ emission for cooling** | **Total CO_2_ emission** |
| 1 | 3450 | 271 | 82.81 | 6.51 | 89.32 | 1.24 | | 0.10 | 1.34 |
| 2 | 3018 | 497 | 72.43 | 11.92 | 84.35 | 1.09 | | 0.18 | 1.27 |
| 3 | 3528 | 229 | 84.67 | 5.50 | 90.17 | 1.27 | | 0.08 | 1.35 |
| 4 | 2421 | 495 | 58.09 | 11.87 | 69.97 | 0.87 | | 0.18 | 1.05 |
| 5 | 3460 | 264 | 83.04 | 6.34 | 89.38 | 1.25 | | 0.10 | 1.34 |
| 6 | 2035 | 807 | 48.85 | 19.36 | 68.21 | 0.73 | | 0.29 | 1.02 |
| 7 | 1641 | 889 | 39.38 | 21.32 | 60.70 | 0.59 | | 0.32 | 0.91 |


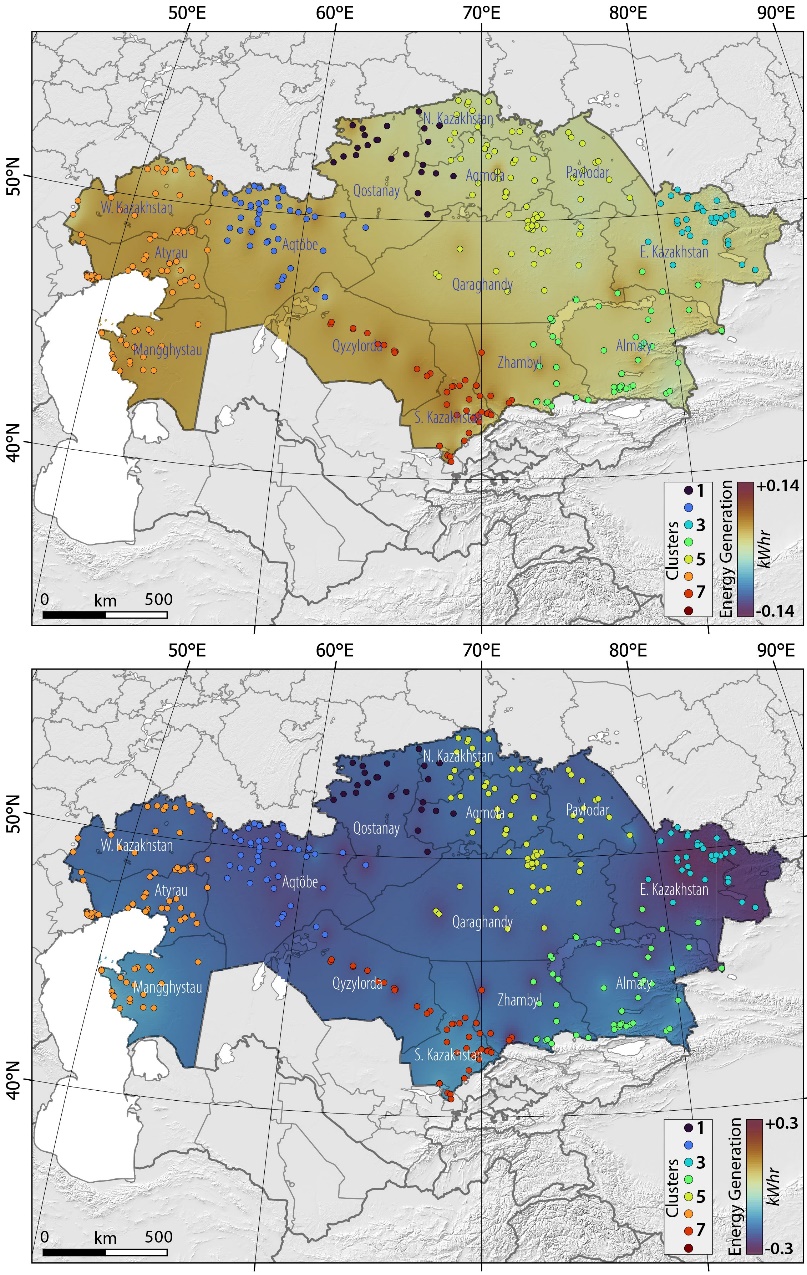


**B**

**A**

**Figure S3.** The slope of spatial-temporal changes in (**A**) Energy generation for cooling (kWh) and (**B**) Energy generation for heating (kWh) in Kazakhstan, Central Asia, between 1959 and 2021. Please note that colored dots and color scale refer to identified clusters and slope of changes, respectively, in Kazakhstan.

## 
